# Virtual patient simulation to improve nurses’ relational skills in a continuing education context: a convergent mixed methods study

**DOI:** 10.1186/s12912-021-00740-x

**Published:** 2022-01-04

**Authors:** Geneviève Rouleau, Marie-Pierre Gagnon, José Côté, Lauralie Richard, Gabrielle Chicoine, Jérôme Pelletier

**Affiliations:** 1 Faculty of Nursing, Université Laval, Pavillon Ferdinand-Vandry, 1050 De la Médecine Ave., Québec City, QC G1V 0A6 Canada; 2 Research Chair in Innovative Nursing Practices, 850 St-Denis St., Tour S, Montréal, QC H2X0A9 Canada; 3 Vitam Research Center in Sustainable Health, Université Laval, 2525 De la Canardière Rd., Québec City, QC G1J 0A4 Canada; 4 Population Health and Optimal Health Practices Axis, CHU de Québec-Université Laval Research Centre, 1050 Sainte-Foy Rd., Québec City, QC G1S 4L8 Canada; 5 Faculty of Nursing, Université de Montréal, 2375 Côte Ste-Catherine Rd., Pavillon Marguerite d’Youville, Montréal, QC H3T 1A8 Canada; 6 Centre de recherche du Centre hospitalier de l’Université de Montréal, 850 St-Denis St, Tour S, Montréal, QC H2X0A9 Canada; 7 Department of General Practice and Rural Health, Dunedin School of Medicine, University of Otago, 55 Hanover Street, Dunedin, 9016 New Zealand; 8 Université du Québec à Rimouski, 300 Allée des Ursulines, Rimouski, QC G5L 3A1 Canada

**Keywords:** Computer simulation, Communication, Nursing continuing education, Motivational interviewing, Mixed method, Nurses, Relational skills, Simulation training, Virtual patient simulation, HIV

## Abstract

**Background:**

Effective provider-patient communication is crucial to the delivery of high-quality care. Communication roadblock such as righting reflex is widely observed among providers and can lead to relational disengagement. In previous work, nurses felt ill-equipped to communicate effectively with HIV-positive patients to support medication adherence. Providing nurses with continuing education opportunities to improve their relational skills is a major target for optimizing the quality of care. Virtual patient simulation is one promising strategy that needs to be evaluated among graduate nurses. This study aimed to assess the acceptability of a virtual patient simulation to improve nurses’ relational skills in a continuing education context.

**Methods:**

We conducted a convergent mixed methods study by combining a quantitative pre-experimental, one-group post-test design and a qualitative exploratory study. We used convenience and snowball sampling approaches to select registered nurses (*n* = 49) working in Quebec, Canada. Participants completed an online sociodemographic questionnaire, consulted the automated virtual patient simulation (informed by motivational interviewing), and filled out an online post-test survey. Descriptive statistics (mean, SD, median, interquartile range) were used to present quantitative findings. From the 27 participants who completed the simulation and post-test survey, five participated in a focus group to explore their learning experience. The discussion transcript was subjected to thematic analysis. At the final stage of the study, we used a comparison strategy for the purpose of integrating the quantitative and qualitative results.

**Results:**

Nurses perceived the simulation to be highly acceptable. They rated the global system quality and the technology acceptance with high scores. They reported having enjoyed the simulation and recommended other providers use it. Four qualitative themes were identified: motivations to engage in the simulation-based research; learning in a realistic, immersive, and non-judgmental environment; perceived utility of the simulation; and perceived difficulty in engaging in the simulation-based research.

**Conclusions:**

The simulation contributed to knowledge and skills development on motivational interviewing and enhanced nurses’ self-confidence in applying relational skills. Simulation holds the potential to change practice, as nurses become more self-reflective and aware of the impact of their relational skills on patient care.

**Trial registration:**

ISRCTN18243005, retrospectively registered on July 3 2020.

**Supplementary Information:**

The online version contains supplementary material available at 10.1186/s12912-021-00740-x

## Background

It widely documented that communication is an essential, core competency for all healthcare providers (HCP), including nurses [1]. Effective HCP-patient communication is crucial to the delivery of high-quality care [2]. Conversely, miscommunication can lead to medical errors, delayed treatment, low treatment adherence, medication errors, patient dissatisfaction, compromised patient safety, and so on [3–5]. One “communication roadblock” that is widely observed among HCP is the righting reflex [6]. The righting reflex is a directive style of communication (or counselling), in which the HCP tries to convince a patient to take an action the HCP thinks is right (such as medication adherence). Although the righting reflex stems from a genuine intention to help, it can compromise the therapeutic relationship and lead to relational disengagement [6]. The findings of our previous qualitative work [7] resonated with this communication roadblock. Indeed, when faced with people living with HIV non-adherence to their medication, nurses provided advice, information, and tips. However, nurses lacked tools to communicate effectively in supporting, convincing, and motivating their patients to “adhere” to their treatment. Providing nurses with continuing education (CE) opportunities to improve their relational skills is a major target for optimizing the quality of care.

Considering the barriers that keep some HCP from attending CE activities, such as staff shortage, cost, travel time, and competing demands [8], the use of virtual patient (VP) simulation in health professions education is a promising training modality to consider. VP simulation can be defined as an interactive, computerized simulation that relies on real-world patient scenarios for the training, education, and assessment of HCP [9].VP simulation makes learning flexible and convenient, as users choose the time and space where training occurs. It can also reduce the use of costly resources, and be disseminated to a broad range of HCP in various settings across far ranging geographical areas [10, 11]. Simulation gives nurses the opportunity to practice with a VP in a safe learning environment where they can make mistakes without causing real patients any harm [12].

Use of VP simulation in health professional training has grown exponentially in the past decade, as many literature reviews in the field have shown [13–18]. Medical education is the most targeted context in these reviews, followed by nursing. When the discipline of nursing is explored, the studies included in the reviews have mainly focused on undergraduate nursing students; the uptake of VP simulation for practicing nurses remains low. Evidence from a meta-analysis show that, compared to traditional methods in pre- and post-registration health-professionals’ education, VP simulation is more effective in improving skills (e.g. clinical reasoning, procedural skills, and mix of procedural and team skills) than knowledge [17]. The use of VP simulation to improve relational skills is documented among students and other healthcare providers [13, 18], but it is less common among graduate nurses in a CE context [14]. Simulation-based learning experience showed a smaller effect size of simulation-based research among staff nurses than among nursing students [19]. One possible explanation of this difference lies in the challenges that nurses experience in the clinical setting (e.g. supplies, time, costs) that could hinder simulation-based research in practice and, consequently, reduce its potential effectiveness. More research is needed to assess the potential of VP simulation among staff nurses in improving their communication skills.

### Aims and objectives

This study aimed to quantitatively and qualitatively assess the acceptability of a VP simulation to improve nurses’ relational skills in a CE context. This study is part of a doctoral dissertation [20]. Acceptability is defined as “a multi-faceted construct that reflects the extent to which people delivering or receiving a healthcare intervention consider it to be appropriate, based on anticipated or experienced cognitive and emotional responses to the intervention” [21].

Our specific research objectives were: a) to measure the extent of the VP simulation nurses’ perceived acceptability in regards of the simulation design elements, of the global system quality and the technology acceptance, of the role simulation plays in supporting nurses’ professional practice, and of the achievement of learning objectives (quantitative objective); b) to explore nurses’ learning experience (qualitative objective); c) to deepen understanding of how the VP simulation contributed to nurses’ uptake of relational skills, to overall learning and its transfer into practice (mixed methods objective).

### Intervention

**Fig. 1:**
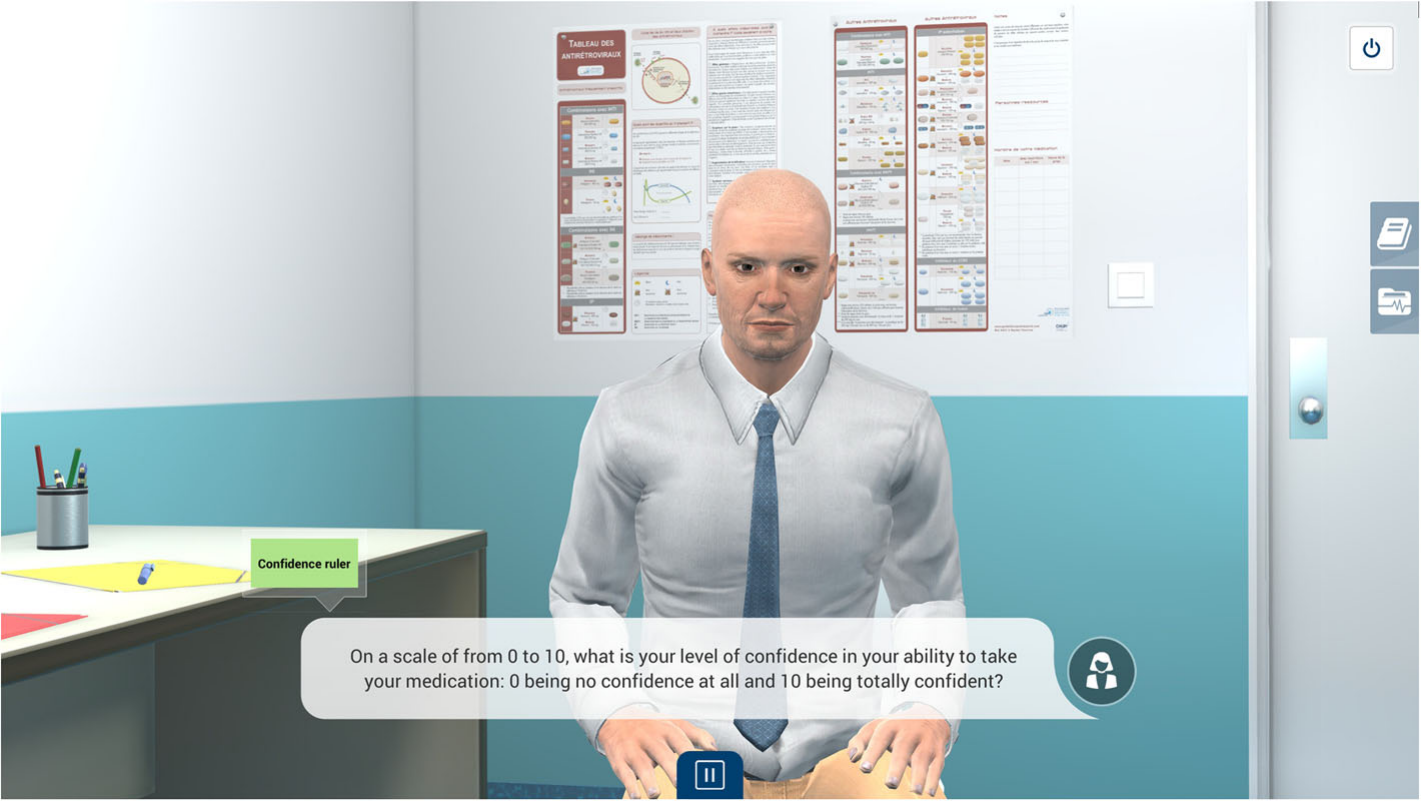
Screenshot of the virtual patient simulation

To fill the gap in this type of educational intervention available for nurses and to address the communication roadblock [7], we developed a VP simulation [22] aimed to improve nurses’ relational skills. The VP simulation was informed by a strengths-based nursing approach [23], motivational interviewing (MI) [6], and adult learning theories [24]. The clinical case scenario is the story of Mr. Wilson, an HIV-positive man having difficulty taking his antiretroviral therapy. The clinical content is informed by MI [6]: MI is  one technique HCP can use to ensure the effective relational skills they need to open up conversation with patients about behavioural changes. MI is defined as a person-centered, collaborative, and guiding communication style (rather than a directive one) that elicits people’s motivation and commitment to change. The core of the simulation is then focused on applying relational skills consistent with MI (e.g., asking open-ended questions, using reflective listening), rather than acquiring HIV-specific knowledge. The screen-based simulation allows users to interact with a two-dimensional animated VP character (see Fig. 1). The simulation includes a prebriefing video and text, an electronic patient record, a glossary, and a simulated nurse-patient consultation. The narrative-simulated consultation encompasses quizzes and feedback loops, in which the learners’ choices and decisions can influence the VP’s speech. Green and red labels were also used as visual and theoretical cues to qualify the nurse-patient dialogue and acted as feedback. Key elements of the simulation are described elsewhere [22]  and are summarized in Additional file 1 in the CONSORT-EHEALTH [25].

## Methods

### Study design

**Fig. 2:**
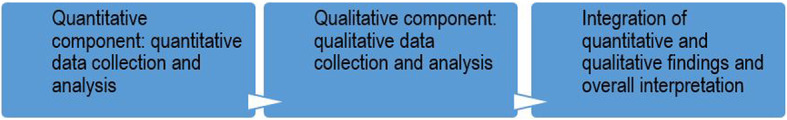
Convergent mixed methods design

We conducted a convergent mixed methods study (Fig. 2). This mixed methods design is defined as the collection and analysis of quantitative and qualitative data integrated for results comparison and complementarity [26, 27]. Both types of data were collected separately and analyzed independently. A comparison strategy was then used to combine quantitative and qualitative results [28, 29], to interpret how the merged results agreed (correspondence, similarities), offered complementary information, or contradicted (disagreement or dissonance) [26]. First, we carried out a pre-experimental study with a one-group post-test design [30] to measure nurses’ perceptions of the VP simulation. Second, we used a qualitative exploratory design [31] to describe nurses’ learning experience and to further nuance and deepen our understanding of the acceptability of the intervention by using complementary topics that were not covered by the quantitative component. The purpose of the integration of quantitative and qualitative findings was to compare and contrast both components to provide a comprehensive picture of the VP simulation’s contribution to nurses’ learning. The samples from the quantitative and qualitative components were interdependent as participants were required to complete the VP simulation and the post-test survey before being invited to take part in the focus group. Each method (quantitative and qualitative) aimed to answer complementary research questions. Some data collection questions targeted the same broad content while using different wordings: the role of the simulation to support nurses’ professional practice (quantitative component) and the concrete implications of having participated in the simulation for nursing practice (qualitative component). Other data collection questions were different such as the acceptance of the technology (quantitative component) and the motivation in participating in the whole study (qualitative component). One qualitative question was formulated from one quantitative statement to get a deeper understanding and explain what was meant for nurses to have gotten a useful learning experience helped to the VP simulation. During the integration phase of quantitative and qualitative findings, we identified content areas represented in both data sets [26] gained from survey and focus group - in order to compare and contrast them.

### Quantitative component

#### Sampling and recruitment

We used convenience and snowball sampling approaches to select registered nurses working in Quebec (Canada) who self-reported having basic computer literacy skills. Convenience sampling is advantageous in terms of affordability and of the immediate availability of the participants. Given the engagement required when participating in an online study, we were looking for nurses who had time and were motivated to complete the whole research process. Nurses were also asked to share the information with their colleagues (snowballing). Snowballing sampling is considered an effective and efficient approach to building a sample through the Internet [32]. We used in-person and online recruitment strategies. We distributed leaflets at a conference involving nurses interested in HIV care and at clinical settings. Information was also communicated via online professional newsletters and e-mail to members of the Quebec order of nurses and of the HIV mentoring program [33]. Table 1 contains a summary describing the nurses’ journey in the research process.

**Table 1:** Nurses’ journey in the research process

Enrollment, intervention and data collection	Activities
Enrollment: pre-intervention and recruitment [March 22–August 5, 2019]	Received online information about the study and the consent form (LimeSurvey) Agreed to previously meet eligibility criteria to get access to the sociodemographic questionnaire: holding a valid nurse’s practice licence (participants had to click this criterion online on LimeSurvey) Filled out online pre-intervention questionnaire, including sociodemographic characteristics, computer literacy skills, MI training, and recruitment strategies (LimeSurvey)
Virtual patient simulation intervention (approximately 45 min) [March 22–August 5, 2019]	Received access to the MedicActiv [34] simulation platform via a secure URL that contained a unique code for the study Created an online account Watched prebriefing video or read scripted text Had unlimited access and exposition to full simulated scenario (including the patient’s electronic record, glossary, and the preprogrammed nurse-patient consultation) during the study period
*Data collection (post-intervention)* *Quantitative component *[March 22–August 5, 2019] *Qualitative component* [September 2019]	Received online post-test survey (LimeSurvey); completion was mandatory to receive a certificate for three hours of accredited CE. Participants who finished all the VP simulation and filled out the post-test survey were qualified as “completers.” The others were called “non-completers (i.e. they completed at least the pre-intervention questionnaire, but did not finish the VP simulation). Participated in an online focus group (voluntary)

#### Outcome measures

We collected quantitative data with LimeSurvey (LimeSurvey Project Team / Carsten Schmitz, 2020) with online post-test survey totalizing 80 closed questions on: 1) VP simulation design elements; 2) global system quality and technology acceptance; 3) role of simulation in supporting nurses’ professional practice; 4) achievement of learning objectives. We used a 4-points Likert scale (1 = Strongly disagree; 2 = Disagree; 3 = Agree; 4 = Strongly agree). We developed the majority of the questionnaire for this study (Additional file 2). Except items from the Technology Acceptance Model [35], which have been used almost entirely in their original form, all other items were inspired from existing tools that we modified to fit our simulation design elements, modality, content, role and learning objectives.

### Virtual patient simulation design scale

This scale development was informed by *Simulation Design Scale* [36] to measure nurses’ perceptions of the simulation design elements (23 items): 1) context of the VP simulation / prebriefing (3 items); 2) glossary (4 items); 3) electronic patient record (1 item); 4) quizzes, feedback, and labels (11 items); 5) fidelity (4 items). Fidelity is the extent to which the VP simulation approaches reality [10].

### Global system quality and technology acceptance

We slightly adapted the French-language version [37] of the *Technology Acceptance Model* [35] to measure these two main dimensions (total: 27 items): 1) global system quality and 2) technology acceptance. Global system quality is divided into the following constructs: system quality (5 items); information quality (3 items); service quality (3 items); and, user interface design quality (3 items). Technology acceptance includes: perceived usefulness (3 items); perceived ease of use (3 items); perceived enjoyment (3 items); and, intention to use (4 items). The higher the score (range of averages: 1–4), the greater the overall acceptance. The reliability of the original instrument [35] is demonstrated by a Cronbach alpha of 0.70 to 0.96 while our adapted version of the scale showed these psychometric properties: 0.68 to 1.00.

### The simulation’s role in supporting nurses’ professional practice

Inspired by a validated French translation [38] of the *Role of Simulation in Nurse Education Questionnaire* [39] targeting the clinical preparation of students graduating from nursing programs, we developed a 22 item-tool. It evaluated the actual and anticipated impact of simulation on the nurse-patient relationship and on nurses’ communication skills, learning, and confidence in their ability to transfer the relational skills into practice.

### Achievement of the learning objectives

This tool was developed to assess nurses’ agreement with the achievement of learning objectives (8 items) following their participation in the simulation.

#### Other questions, measures, and data

Open-ended questions were asked in the post-test survey to gain complementary insights about: a) the VP simulation design elements (e.g. comments about the electronic patient record, fidelity); b) the achievement of additional learning objectives; c) the most and least appreciated elements of the VP simulation; d) recommendations to improve the simulation.

The pre-intervention questionnaire included information on nurses’ sociodemographic characteristics, such as age group, gender, education level, workplace setting, previous MI training, and computer literacy skills.

#### Sample size

A total of 30 participants was targeted to take part in the simulation and fill out the post-test survey. This sample size was determined according to recommendations for pilot studies [40, 41], considering that acceptability is often an element that is assessed in these type of studies [42].

#### Quantitative data analysis

A descriptive statistical analysis was conducted using Microsoft Excel version 2013 (Microsoft Corporation). Means (m), standard deviations (SD), median (med), and interquartile range (IQR) were calculated for continuous variables as well as for counts and percentages for the categorical variables. Fisher’s exact test was performed to compare the proportions of participants’ characteristics at baseline between completers and non-completers. This statistical test is justified when the sample size is small [43].

### Qualitative component

#### Data collection

The focus group used a semi-structured conversational approach. It aimed to describe in greater detail and further nuance participants’ experience of the VP simulation as well as deepen our understanding of particular quantitative items, such as the utility of the VP simulation. The questions used to guide the focus group (Table 2) covered these topics: motivations, perceived difficulties for study participation, and concrete implications for nursing practice. The data collection was performed online through synchronous interactions using the Zoom videoconferencing platform (Zoom Video Communications Inc., 2016). The discussion was recorded after receiving participants’ consent.

**Table 2:** Examples of questions used to guide the focus group

• I’d like to hear about what led up to your participation. ° How did you hear about the project? ° What motivated you to take part? ° How did you get the idea of participating in the simulation?	
• In the survey you filled out, everyone agreed or strongly agreed that participating in the virtual simulation was a useful learning experience for their ongoing professional development. How was the simulation useful in your respective work contexts? What did you gain from it?	
• What are the strengths of this simulation? What are its weaknesses or areas that could be improved?	
• In your opinion, what could explain why some people did not finish their participation in the simulation? What difficulties did you yourself encounter?	
• What tangible effects did your participation in the simulation have on your practice? What do you take away from this training activity?	

#### Qualitative data analysis

The focus group recording was transcribed verbatim. Qualitative data analysis followed an inductive and iterative process. We thematically analyzed narratives from the focus group [44, 45]. Coding was led by GR and involved comparison across transcripts. The team members were involved in discussions of preliminary thematic findings and throughout the data interpretation process. NVivo Software Pro 12 (QSR International Pty Ltd., 2018) was used to facilitate data management and organization.

Credibility as a source of trustworthiness [46, 47] was ensured through prolonged engagement and observation. GR was immersed in the data by being directly involve in participant recruitment, data collection (leading the focus group) and analysis. GR listened the audio recording and transcribed the verbatim. She encouraged participants to express themselves freely, by sharing their true impressions of the VP simulation and not what they thought the student-researcher wanted to hear (in order to reduce the desirability bias). Peer debriefing was another way of ensuring the credibility, by discussing the preliminary qualitative findings as well as the mixed method interpretation findings with the team having expertise in qualitative and mixed methods research. A detailed and accurate descriptions of the sociodemographic characteristics of the nurses, the recruitment strategies and the settings was provided as a way of enhancing the transferability of the findings to other similar population and contexts.

### Mixed methods integration

**Fig. 3:**
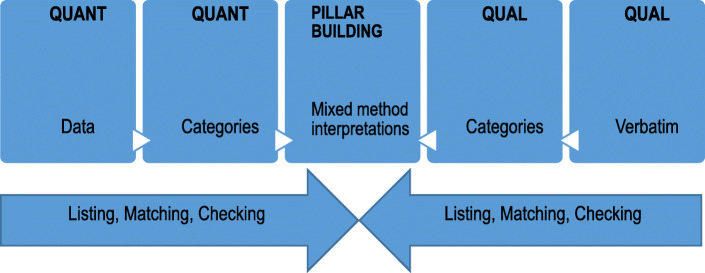
Pillar integration process, adapted from Johnson et al. [28]

Once quantitative and qualitative data were collected and analyzed separately, both components were integrated using a comparison of results strategy [48]. We first used a weaving technique, inspired by Fetter et al. [49, 50], that aims to narratively group both quantitative and qualitative findings under a mixed methods interpretation. For this interpretation process, we utilized the four stages of the pillar integration process (Fig. 3) [28] to visually compare quantitative and qualitative components and integrate them into a joint display (Additional file 3).

We reported the quantitative component, including the simulation description, with CONSORT EHEALTH [25] (Additional file 1). We used CHERRIES for Internet e-surveys [51] (Additional file 4); COREQ for the qualitative component [52] (Additional file 5), and GRAMMS for the mixed methods study [53] (Additional file 6).

## Results

### Participants’ flow chart and characteristics

**Fig. 4:**
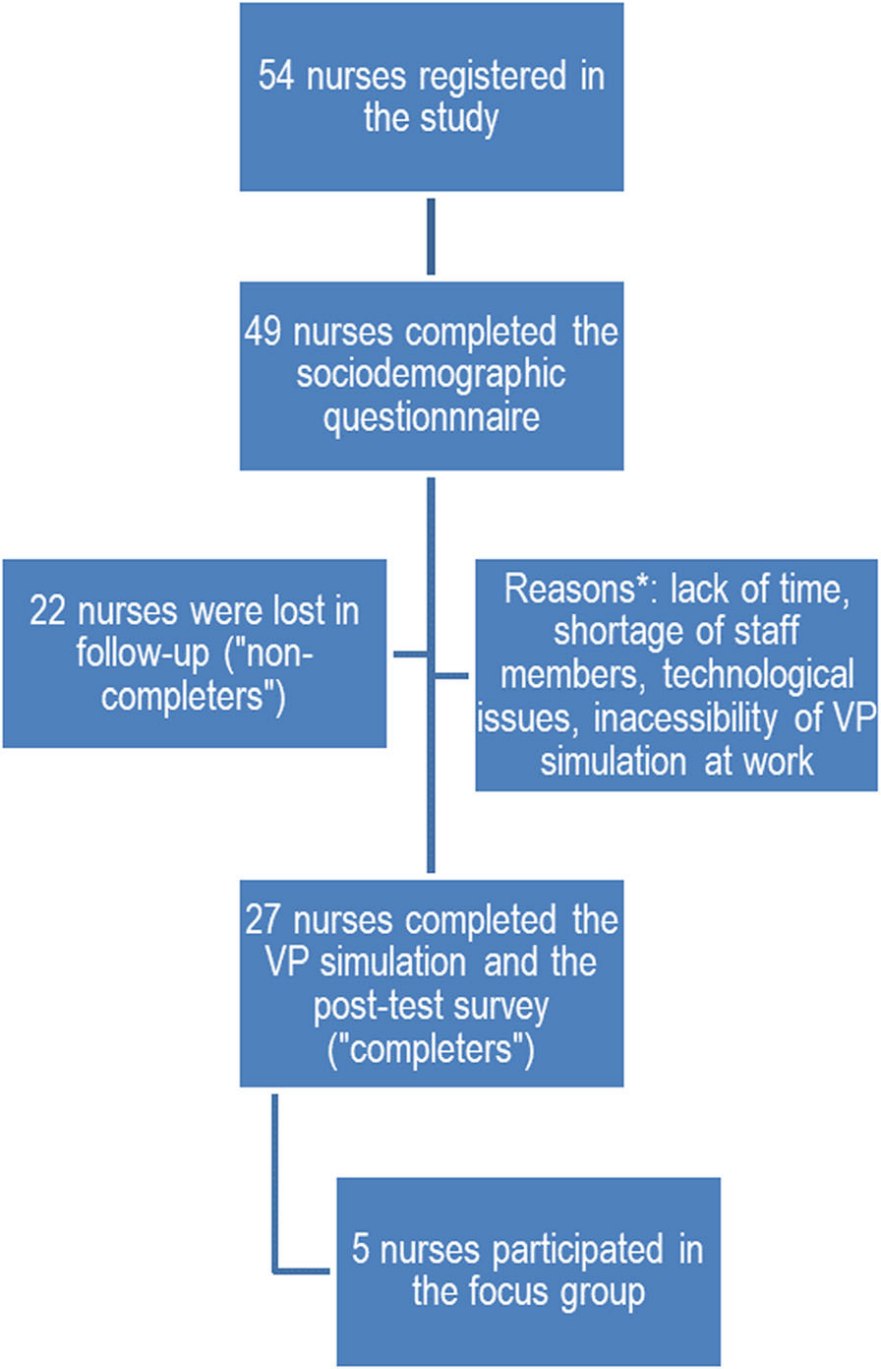
Flow chart of the completers and non-completers. Legend *: The student-researcher and most of the participants kept in touch via e-mail during the research period. Reminders were sent to participants to invite them to complete the VP simulation. During asynchronous e-mail communications, some participants indicated the reasons for not completing the study

The participant flow chart of completers (*n* = 27) and non-completers (*n* = 22) is presented in Fig. 4.

Most of the completers held a bachelor’s degree. They had been working as nurses for an average of 18 years. Eighteen nurses (66.67%) had experience as HIV nurses. Eight nurses (30%) had previous MI training. Majority of participants (25/27, 93%) reported being confident in their computer skills.

From these 27 completers, five nurses took part in the qualitative component: two men and three women. They worked with different clienteles, including people living with HIV (PLHIV). Four nurses were trained in MI. Additional completers and non-completers’ characteristics are presented in Table 3.

**Table 3:** Nurses’ sociodemographic characteristics, computer literacy skills, MI training and recruitment strategies

Characteristics	Completers (***n*** = 27)	Non-completers (***n*** = 22)	***p*** value^a^	Focus group (***n*** = 5)
**Age group, n (%)**			0.89	
25–34	7 (25.93)	5 (22.73)		1 (20.00)
35–44	8 (29.63)	7 (31.81)		0 (0.00)
45–54	8 (29.63)	5 (22.73)		4 (80.00)
55 and over	4 (14.81)	5 (22.73)		0 (0.00)
**Gender, female, n (%)**	22 (81.48)	18 (8.82)	0.74	3 (60.00)
**Education levels, n (%)**			0.58	
Associate’s degree	3 (11.11)	5 (22.73)		0 (0.00)
Certificate/ Bachelor’s degree	19 (70.37)	13 (59.09)		3 (60.00)
Specialized graduate diploma/Master’s degree/PhD	5 (18.52)	4 (18.18)		2 (40.00)
**Employment, n (%)**			0.06	
Full time	19 (70.37)	20^b^ (90.91)		5 (100.00)
Part time	8 (29.63)	1(4.55)		0 (0.00)
**Title, n**^**c**^ **(%)**			0.56	
Nurse-clinician	14 (48.28)	8 (32.00)		2 (33.32)
Nurse	4 (13.79)	7 (28.00)		0 (0)
Research nurse	4 (13.79)	2 (8.00)		0 (0)
Assistant head nurse/head nurse	2 (6.90)	4 (16.00)		1 (16.67)
Professor	1 (3.45)	2 (8.00)		1 (16.67)
Researcher	1 (3.45)	0 (0.00)		1 (16.67)
Other^d^	3 (10.34)	2 (8.00)		1 (16.67)
**Years of practice as nurse, mean (range)**	18.37 (1–42)	18.59 (3–37)		23(8–32)
**Quebec area, n (%)**			0.77	
Montreal	14 (51.85)	13 (59.09)		4 (80.00)
Outside Montreal	13 (48.15)	9 (40.91)		1 (20.00)
**Experience as HIV nurse, n (%)**			1.00	
No	9 (33.33)	8 (36.36)		2 (40.00)
Yes	18 (66.67)	14 (63.64)		3 (60.00)
**Years of practice as HIV nurse** **Mean (range)**	9.87^e^ (0.17^f^ − 23)	6.92^g^ (1–19)		13.5 ^h^ (4–23)
**Previous MI training, n (%)**			0.75	
I don’t know	0 (0.00)	1 (4.55)		0 (0.00)
No, I haven’t received training	17 (62.97)	12 (54.55)		1 (20.00)
No, I haven’t received training, but I have done self-training (autodidact)	2 (7.40)	3 (13.63)		1 (20.00)
Yes	8 (29.63)	6 (27.27)		3 (60.00)
**Previous experience with VP simulation, n (%)**			0.72	
No	26 (96.30)	20 (90.90)		5 (100.00)
Yes	1 (3.70)	1 (4.55)		0 (0.00)
Don’t know	0 (0.00)	1 (4.55)		0 (0.00)
**Confidence in using technology, n (%)**			0.82	
I do not at all feel confident in my skills	0 (0.00)	0 (0)		(0.00)
I feel somewhat confident in my skills	2 (7.41)	1 (4.55)		(0.00)
I feel confident in my skills	13 (48.15)	13 (59.09)		(0.00)
I very feel confident in my skills	12 (44.44)	8 (36.36)		5 (100.00)
**Participation in this web-based research is stressful, n (%)**			0.60	
Strongly disagree	15 (55.56)	10 (45.45)		4 (80.00)
Disagree	11 (40.74)	11 (50.00)		1 (20.00)
Agree	1 (3.70)	0 (0.00)		0 (0.00)
Strongly agree	0 (0.00)	1 (4.55)		0 (0.00)
**Recruitment strategies, n (%)**			0.80	
In person ^i^	16 (59.26)	11 (50.00)		5 (100.00)
HIV mentoring program	6 (22.22)	5 (22.73)		0 (0.00)
Quebec order of nurses	5 (26.32)	6 (27.27)		0 (0.00)

^a^ The p value was calculated with Fisher’s exact test

^b^ One person indicated “retired”. We considered it as a missing value in the Fisher’s exact test calculation

^c^ The *n* per category of participants is calculated by the total numbers of responses instead of the sample size, because some participants indicated more than one title. Completers indicated 29 responses, the non-completers, 25, and the participants of the focus group indicated 6 responses

^d^ Pharmaceutical representative, senior advisor/specialized clinical analyst, manager, nurse practitioner

^e^ 4 missing values

^f^ 0.17 year: 2 months

^g^ 3 missing values

^h^ 1 missing value

^i^ Nurses heard about the project through student-researcher or by coworkers

### All participants’ recruitment strategies

Participants were recruited in person (28/49, 57.14%), i.e. by being informed by a colleague or by the student-researcher; by e-mails sent by the Quebec order of nurses (11/49, 20%) and the HIV mentoring program (10/49, 20%).

### Quantitative findings of completers

The detailed quantitative findings are presented in the additional files: the simulation design elements (Additional file 7), the global system quality and technology acceptance (Additional file 8), the role of the simulation (Additional file 9), and the learning objectives achievement (Additional file 10). Highlights are presented in each subsection.

#### Simulation design elements

A great majority (93%) of participants watched the video content and 78% read the corresponding text on the context of the simulation. Most of the participants (89%) felt that, to understand this context, it was key to have access to both text and video.

All participants agreed that the labels constructively supported their learning. Some 96% found that these cues were key to qualifying the content of the nurse-patient dialogue. All participants agreed that quizzes made them reflect on their nursing practice and they saw themselves in the quiz answers.

Almost all participants (96%) agreed that the feedback was provided in a timely manner (i.e. as the consultation progressed). All participants agreed that the feedback allowed them to make connections between the simulated situations and the theoretical elements of MI.

A majority of participants agreed with the simulation’s fidelity: the patient’s story (96%), the HIV-positive man’s appearance (96%), the nurse-patient interactions (93%), and the nurse’s office (85%) were all perceived as authentic.

#### Global system quality and technology acceptance

The mean score was rated a 3.65 (±0.48) for the *service quality* construct among participants who used the VP simulation support services (11/27). The *interface design quality* (3.54 ± 0.55) was the second construct with the highest score of the global system quality dimension, followed by *system quality* (3.51 ± 0.54) and by *information quality* (3.49 ± 0.50).

Participants had a good *intention to use* (3.53 ± 0.60) the VP simulation. Nurses *perceived enjoyment* (3.47 ± 0.57) and an *ease of use* (3.42 ± 0.67) with the simulation. The lowest mean score of the technology acceptance dimension was the *perceived usefulness* (3.35 ± 0.71), which is, above all, highly acceptable.

#### The role of simulation in supporting nurses’ professional practice

The items with the highest scores were: simulation led nurses to reflect on their practice in general, not just with PLHIV (3.58 ± 0.58); the content will lead nurses to improve their communication skills with clienteles other than PLHIV (3.50 ± 0.51), the health of PLHIV (3.50 ± 0.51), and the quality of therapeutic relationships with PLHIV (3.50 ± 0.51).

#### Achievement of learning objectives

Scores on the achievement of objectives ranged from 3.35 to 3.58, indicating a favourable assessment by participants. These two learning objectives had the highest scores: identification of traps within nursing interventions that can shut down communication with the patient (3.58 ± 0.50), and those that can optimize openness to the patient’s experience (3.54 ± 0.51).

### Qualitative findings

Four main themes are presented: 1) Motivations to engage in the simulation-based research; 2) Learning in a realistic, immersive, and non-judgmental environment; 3) Perceived utility of the simulation; and, 4) Perceived difficulty in engaging in the simulation-based research.

#### Motivations to engage in the simulation-based research

Participants identified several reasons for taking part in the simulation-based research. First, the simulation offered accreditation and was free of charge, which were appealing incentives. Second, nurses reported that their interest and curiosity had been stirred by the learning modality, which was perceived as innovative, stimulating, and interactive, and by the way MI could be transposed into technology:

I was curious to see this new training modality because I have already followed MI training, and sometimes we’d practice with a coworker. I was curious to see how far we could get with the simulation. (Female nurse-manager)

Nurses perceived that the simulation could be applicable and coherent in their own practice with different clienteles (e.g. youth, people with hepatitis C), and, more broadly, to a variety of contexts:

[The simulation] was addressing the issue of adherence to HIV treatment and I felt that [the topic] fit in well with my practice. (Male assistant head nurse)

I thought [the simulation] was something that was interesting and not just about HIV [...] it was something that could be transferred to other areas of activity. (Female school nurse)

Finally, the desire to learn new knowledge or strengthen existing knowledge about MI and HIV were factors motivating nurses’ participation.

I found it important to do this training to learn things about HIV but also about motivational interviewing, which we do daily, enormously, at our office. (Female school nurse)

#### Learning in a realistic, immersive, and non-judgmental environment

Two nurses who were experienced in providing HIV care reported the VP’s story to be an uncommon one for non-adherence, but felt that it was nonetheless credible and realistic. What they felt to be most important was the nurse-patient interaction, which allowed to immerse themselves in the simulation:

Maybe this is because I’ve done a lot of work around the issue of taking antiretroviral treatment, so I found the [VP’s] situation ... maybe less typical... At the same time, I realized that it was not necessarily very important. Eventually, you forget about the situation, you know, because [the learning activity] is more about how to react to interactions with the patient [...] I was more focused on what he was saying than the image. I think it’s a really strong point of [the learning activity] that we got really into it. (Female nurse-researcher)

One nurse’s first impression was the VP’s resemblance to a puppet, which lead him to wonder about the seriousness of the learning activity. The patient’s appearance could have caused this participant to lose interest in the learning experience, but eventually this image of the VP gave way to a more human and realistic impression:

At first, I thought [the VP] looked like a puppet [...] I kind of wondered if [the simulation] was for real. I don’t really want to question its seriousness … Beyond the caricature, I could see the patient asking himself questions; he was squinting a little. Human beings do that. They’re not puppets [...] And as I went along doing the interview, I saw there was communication between the nurse and the patient. And [my impression] faded away. (Male nurse case-manager)

Two participants compared the simulation to physical presence-based group learning, where MI must be practiced through role-playing with a coworker. The simulation was seen as an advantageous way to reproduce a real interaction with a VP, reducing the discomfort and bias of practicing with someone, and fostering the learning progress:

In classic training activities, we practice with a coworker. I find that quite biased because we’ve both just learned the theory; we try to apply it; the other person has just learned the same thing so, in the end, well, we help each other only a little bit. But here, we were faced with a virtual character who is very realistic. I find it even more real than with, shall we say, another trainee. But for people who are shy in groups, [the simulation] is really very accessible and allows them to progress. (Male assistant head nurse)

Compared to group training activities, the simulation provides freedom while targeting individual learning and performance:

I think that doing it one by one, well, alone, allows something that is not necessarily possible in a group training activity. It’s even more in-tune with what you would actually do. There is no judgment. There are no right or wrong answers. [The simulation] allows you to answer more freely. (Female nurse-researcher)

Finally, this participant summed up her experience: *“I feel like I got real practice.”* (Female school nurse).

#### Perceived utility of the virtual patient simulation

We identified three sub-themes as part of this theme: developing reflective learning and transferring it to practice, being present and revisiting relational skills, and acquiring and consolidating motivational interviewing knowledge and skills.

### Developing reflective learning and transferring it to practice

All the nurses mentioned the simulation’s capacity to promote mistake-based learning through quizzes and feedback loops:

It was fun because it’s like action/reaction. It was immediately obvious if you asked the question wrong, you could see the effect. I found it interesting because if you took a wrong action, you could get back on track. That way, we could understand why it was a mistake. (Female nurse-manager)

This participant, who did the entire virtual simulation twice, reported a progression of his learning, building on the mistakes he had made:

The first time, I made a lot of mistakes because I told myself that I was going to go with my knowledge and experience. The second time, I did it with my new knowledge. It gives you parallel vantage point onto yourself, onto your own beliefs. (Male nurse case-manager)

The simulation thus allowed participants to reflect and take a critical look at themselves and their practice, becoming aware of past mistakes and the impact of their interventions on their relationship and interactions with patients:

You’re never neutral in a MI. Yes, you’re the care provider, but you’re a person. It can set certain limits or can even make you get stuck in it. [The simulation] makes you aware of who you are through all this. (Male nurse case-manager).

Look, if patients don’t react or aren’t motivated, well, maybe it’s because I too am playing a part as the care provider: maybe I am not addressing them in the right way, maybe I am not considering them in their entirety, according to their beliefs and values. (Female nurse-manager)

The interactivity inherent to the simulation supports this reflexive process, which in turn can lead to transferring learning to real practice, and thus improve it:

When you’re one-on-one [with a young person], sometimes you’ll answer off the cuff because you’re in a hurry. If you’ve practiced [the situation] in simulation, you’re going to know that whatever you said was not so great, you know, you’re going to question yourself. So, you’re going to be more careful when a similar situation occurs in reality [...] I’m going to try saying it differently to help the person get a little further. It makes you better. (Female school nurse)

This participant questioned his past interventions, in which he hastily presumed the cause of non-adherence (e.g. relapse, substance abuse) when interacting with his clientele. After participating in the simulation, this nurse stated his intention of changing his way of intervening so that he better understands the patient’s situation, before drawing conclusions:

Do I go too fast sometimes? Telling myself that, well, he didn’t take it [his treatment], that he must have relapsed, always jumping to my conclusions first. Don’t I miss things sometimes, too? I was thinking that maybe now I will be more careful and try to understand the patient’s reasons and stop just saying ‘Ah, well, he didn’t take it.’ (Male assistant head nurse)

### Being present and revisiting relational skills

The simulation helped to underscore the importance of listening to patients. This meant being present, available during the consultation and living in the “here and now”:

It helps nurses understand or realize that it’s important to listen, to be there in the here and now. More and more, we have our electronic medical records, we write in the record and don’t even look at the patient. We no longer take the time to actually look at the patient because we are so busy on our computer... It’s really worth it to sit down and look at the patient and just be present with them. (Female nurse-manager)

The simulation had a positive influence on revisiting ways of communicating and asking patients the right questions to support them in reflecting and identifying their own solutions:

I’d say it’s more in the way the questions are asked. It’s really focused on open-ended questions, and solutions that come from the patient. We [nurses] may have solutions, but they have to come from them [the patients], and that’s when they are most effective [...] How can we ask questions that bring out the best in the patient? (Male nurse case-manager)

The simulation alerted the nurses and raised their awareness of how they relate to patients, creating optimal conditions for successful relational practice and mobilizing communication skills that allow patients to express themselves and, especially, to find their own solutions.

### Acquiring and consolidating motivational interviewing knowledge and skills

One participant with no prior MI training considered the simulation to be an effective and efficient way to achieve intensive learning:

I’d read a little about MI, but I’d never done any training. I didn’t expect to learn so much in such a short time. (Female nurse-researcher)

Moreover, for another participant, who had received training in MI and who does not practice directly with patients, the key lays in putting theoretical elements into action with the VP. Consequently, the simulation-facilitated practice helped reinforce her knowledge and feelings of competence in applying MI:

I had already had some MI training. [The simulation] reassured me a bit that, actually, I was competent and that I would have been good, face-to-face, with a patient. So, it just confirmed this for me. Because there’s always a doubt about MI being this huge thing. But in the end, you know, we just lack practice. And I found that the platform meant that I was able to strengthen my nursing practice and my past theoretical learning, since I don’t see patients every day. (Female nurse-manager)

For the other three participants who had previous MI training, the simulation helped them better understand the theory and refresh their knowledge, as well as learn how to better apply it. Simulation as a learning modality thus seemed to benefit nurses with various levels of MI training and knowledge.

#### Perceived difficulty in engaging in the simulation-based research

We asked participants in the focus group to reflect on the difficulties they experienced in completing the study, or those they heard their coworkers mention. Technical difficulties were noted as one of the main potential explanations of some participants’ withdrawal, either because of the complexity of creating an account, the delay between the characters’ words and movements, or the system’s slowness. Individual perseverance became important in this context:

I’m not saying the workflow was slow... but maybe that’s why some people didn’t finish the training activity. I’m not saying it was repetitive, but maybe if they feel it was too slow... When the patient talks, he moves his arms around, and sometimes there was a little delay. This was maybe a feeling I had, since I was persistent at first. (Female nurse-manager)

One participant did not like the simulation’s lack of progress indicators, which she felt might also have discouraged others. Individual and time-related elements were another hypothesis for some participants’ withdrawal:

Perhaps a lack of time or a drop in motivation along the way. When I start something, I like to finish it. So maybe it’s question of personality, too. (Female school nurse)

### Integration of quantitative and qualitative findings into mixed method interpretations

Using the pillar integration process (see Additional file 3), we have combined both quantitative and qualitative data and categories (findings). The operationalization of this process as well as an excerpt of the joint display that provides a side-by-side comparison of both types of data and categories is presented in Additional file 11. Examples are provided to support the identification of the four mixed method interpretation findings resulting from our analysis: 1) Influence of the simulation’s fidelity on nurses’ impression of getting a real practice and of having an immersive learning experience; 2) Simulation’s perceived flexibility, efficacy, and control over one’s learning led to a positive learning experience; 3) Taping self-awareness and reflection in relational practice, 4) Acquiring new knowledge and building self-confidence. These findings describe how the VP simulation quality, its element designs and its role contributed to nurses’ learning experience.

#### Influence of the simulation’s fidelity on nurses’ impression of getting a real practice and of having an immersive learning experience

While various simulation design elements were assessed quantitatively as being realistic, the qualitative results provide insight into how fidelity contributed to nurses’ immersion in their learning experience, among other gains. The quantitative and qualitative results are therefore complementary. Participants were able to overcome the VP’s appearance and become immersed in the scenario to focus on the nurse-patient interactions. They also felt the simulation gave them an opportunity for real practice.

#### Simulation’s perceived flexibility, efficacy, and control over one’s learning led to a positive learning experience

As described in Additional file 8, global system quality and technology acceptance were rated with high scores. VP simulation offers flexibility for when and where learning occurs, it gives users control over their learning, and it was generally perceived as more effective than other types of training. These aspects all positively influenced participants’ learning experience. The qualitative findings supported the quantitative results. All participants in the focus group appreciated being able to use the simulation during or outside of work hours and even from home. The flexibility of the learning modality allowed them to consult the simulation more than once. Compared to face-to-face training that requires trainees to practice with a colleague, the simulation gave them practice with the VP that was both more realistic and less intimidating. This modality therefore allows users to express a sincere response, without fear of making a mistake in front of a group. Simulation also facilitates the evaluation of individual knowledge and performance, rather than collective ones.

#### Taping self-awareness and reflection in relational practice

The quantitative results indicate that the high scores in favour of the role of simulation, quizzes, and feedback prompted the participants to reflect on their nursing practice, make connections between theory and practice, learn from mistakes, and raise their awareness of elements that can facilitate or hinder therapeutic relationships with patients. The qualitative results also enriched the quantitative results when nurses gave concrete examples of their own communication styles that had been less effective in the past (e.g. leading the consultation, making recommendations to the patient without asking permission, jumping to conclusions too quickly) and that could be improved. The nurses said that practicing with the VP and getting synchronous feedback that mirrors their actual practice would help them avoid replicating ineffective patterns. The simulation therefore contributed to educating and raising awareness of self, as nurse, and of others (i.e. patients), and underscored the importance of nurses’ presence, open-mindedness, availability, and good listening.

#### Acquiring new knowledge and building self-confidence

By assessing the *role of simulation in supporting nursing practice*, participants reported having learned something new. They also expressed having built self-confidence. Indeed, they felt capable of applying communication skills and of facing similar situations with PLHIV and other clienteles in the future. The qualitative results reinforce these findings, reflecting the simulation’s influence on nurses feeling better prepared and equipped to apply MI with their clienteles, to consolidate their practice, and thus to reinforce their sense of confidence and competence.

## Discussion

### Statement of main findings

Overall, nurses perceived that VP simulation is highly acceptable, if we consider that the great majority of means were above 3, on the 4-point Likert scale. The quantitative results were highly consensual in favour of simulation design elements, global system quality and technology acceptance, the simulation’s role in supporting nursing practice, and the achievement of learning objectives. The qualitative results nuanced, deepened, and even added new elements (e.g. motivation to participate and difficulties encountered) to the quantitative results. The integration of quantitative and qualitative findings drew a full portrait of the continuum of the nurses’ simulation-based experience, the VP elements that contributed to their immersive learning, and its potential transfer to their practice. Four mixed method interpretations were described: 1) Influence of the simulation’s fidelity on nurses’ impression of getting a real practice and having an immersive learning experience; 2) Simulation’s perceived flexibility, efficacy, and control over one’s learning led to a positive learning experience; 3) Taping self-awareness and reflection in relational practice; 4) Acquiring new knowledge and building self-confidence.

### Comparison with existing literature

The qualitative theme *motivations to engage in the simulation-based research* brings a new element to the quantitative results, which could not capture this perspective. We created and reinforced what Moore et al. [54] call a *teachable moment* in order to influence the enrollment, participation, and engagement of nurses in this learning activity. To do so, we shared information about the project via different channels, with a view to reaching a variety of nurse profiles. The majority of nurses who completed the VP simulation was recruited through in-person strategy (16/27). The *social influences*, such as recommendations by colleagues, are recognized as facilitators to the self-directed learning of physicians in CE, alongside with affective attitude (e.g. professional interest, motivation, willingness) and training accreditation [55]. Learners’ characteristics/qualities, like perseverance and the desire to finish what has been started, are also considered to be elements that can affect both learning and the motivation to engage in the simulation [36, 56].

VP simulation led to immersive, realistic, active, and constructive learning experiences. Prebriefing was planned in the VP simulation codevelopment process [22] and is considered a best practice [57, 58]. Prebriefing is known as a facilitation method and a preparatory activity to ready learners for the simulation-based experience. The red and green labels were cues that served as feedback, and were also used as a facilitation method to orient learners through the simulation [57].

Feedback is considered to be the most important feature of effective learning [16, 59]. Indeed, it can support learners’ self-assessment of their skills and allow the progression, development, and maintenance of those competencies [59]. In the quantitative results, participants reported appreciating the timing of the synchronous feedback. The timing of feedback can indeed play a role in learning, as can its source (how it is provided, by whom) and type (i.e. outcome or process-based). One other meaningful feature of the simulation is the opportunity of deliberate and repetitive practice, which can impact learning [59, 60], unlike other educational interventions, such as conferences or lectures in which learners are often passive recipients of “inert” information [61].

In light of our results and when comparing with the literature, it is reasonable to believe that different modes of fidelity, be it physical, conceptual, emotional/experiential [62, 63], not only affected participants’ learning, but also their engagement, overall experience, and immersion in the simulation [59, 62–64]. Physical fidelity refers notably to the VP’s appearance and the nurse’s virtual office. In the VP simulation, conceptual fidelity is illustrated by the if/then concept [62, 63]: *if* the learner adopts a relational skill consistent with MI (e.g. a guiding style of counseling vs a directing one), *then* it will open the dialogue with the patient. One participant clearly expressed this mode of fidelity by the “action/reaction principle.” Finally, participants in the focus group reported the sense of having truly practiced; they found that simulation was an effective learning approach, reflecting the emotional and experiential mode of fidelity [62]. This mode refers to the learner’s emotions, feelings, and beliefs relating to their entire experience of participating in the simulation [62, 63].

It would seem that the benefits of the simulation extend beyond the learning of the recommended communication techniques to encompass a relational component: being present, taking the time to listen carefully and understanding patients without falling prematurely into professional preconceptions. This relational component is aligned with partnership, one of the vital aspects of MI [6] that has to do with seeing the world through the patient’s eyes without imposing the nurse’s view. Another important finding of this study is the reflective learning developed during nurses’ simulation-based experience. Reflection was indeed central to the learning experience because it played a double role, as both a reflective methodology (i.e. synchronous feedback used to promote reflection and to learn from one’s mistakes) and a learning outcome [65] (i.e. VP simulation allowed nurses to develop reflective learning). Reflection is considered to be a means of supporting professional development [66] and has the potential to transform experience into new learning [67]. A meaningful finding of our study is that nurses raised their awareness of themselves, and of others (i.e. patients), by acknowledging how some elements (such as their own communication styles) could influence positively or negatively therapeutic relationships with patients. Perceptual skills have been reported as a communication skills learning outcome in a qualitative synthesis (*n* = 168) targeting health professions workers [1]. Perceptual skills (self-awareness, awareness of others and context) accounted for 9% of learning outcomes throughout the whole papers and were considered of importance in reflective, self-regulating health providers. Bennett-Levy [61] sheds light that interpersonal perceptual skills are fundamental to effective therapeutic practice, and include these three attributes: empathy, mindfulness and reflection-in-action. By bringing into the table these notions of reflection and perceptual skills, in agreement with Denniston et al. [1], we argue that relational skills are complex. Training interventions aimed to address relational skills, including communication between healthcare providers and patients, should consider an array of relational skills learning outcomes in their evaluation design.

Our findings also corroborate those of a qualitative studies [11, 68] and those reported in an integrative review (*n* = 38 articles) [18] that explored how VP simulation influenced the non-technical skills (such as communication) of undergraduate nursing students [11] and undergraduate health professional education [18]. The findings suggest that students acknowledged the importance of communication and listening to their patients. The VP “opened their eyes” to the impacts either effective or poor communication had on healthcare [18]. VP simulation exerted a positive influence on students, by reinforcing or teaching new communication skills, providing opportunities for practicing those skills and for building their confidence in applying them, developing specific verbal and nonverbal communication skills [11] and developing awareness of non-technical skills including but not limited to communication [68].

### Strengths and limitations

As far as we are aware, this is the first study to examine the acceptability of a VP simulation informed by MI to improve nurses’ relational skills in a CE context. This mixed methods study led to a gain in complementary and rich data, providing a comprehensive picture of nurses’ learning experience. However, two main challenges were encountered during the integration. The first was dealing with huge numbers of items and data in the quantitative survey (*n* = 80 items) and comparing these to the qualitative data that was focused on circumscribed thematic. The second was the unequal sample size (27 in the quantitative component; and 5 in the qualitative part). Use of the joint display was helpful in putting together data and results that can be compared and discussed. Limitations of one method associated with the present of the other method are described in further details in Additional file 6.

The convenience sampling and snowball approaches used in both quantitative and qualitative components allowed us to recruit nurses with various profiles who work in different settings, thus adding to the richness of the findings. However, those sampling approaches have their own limitations. The findings may have been tainted with participation bias, given our sample had to complete 100% of the simulation and the post-test survey. It means that only the volunteers and the most “motivated” and willing nurses participated. This could explain the consensual findings in favour of the VP simulation. It would have been useful to also explore the reasons why nurses abandoned the simulation; but this was unfortunately not possible. The nonrepresentativeness of this captive sample of nurses may have limited the transferability of the findings to other contexts and population [69]. In addition, the five-participant sample size was relatively small (focus group).

An important limitation of our study is that we did not follow the best practices for scale development and validation, such as those suggested by Boateng et al. [70]. This is consistent with the exploratory lens of our study. With the exception of the Global System Quality and Technology Acceptance instrument [35], the other tools were not validated. However, to ensure content clarity and ease of navigation, we conducted two rounds of survey pre-tests with potential end users before launching the study.

### Implications for research

The variety of the nurses’ profiles provided insight into the transferability of the VP simulation beyond the field of HIV care. In the post-test survey, nurses recommended the simulation to other healthcare professionals. Further research could focus on simulation acceptability for a variety of providers. Subsequent research would be necessary to explore the influence of contextual enablers/barriers (e.g. resources, structure, organizational culture) and those that are related to healthcare professionals themselves (e.g. role, work structure, habits, competing demands) when using VP simulation to apply the resulting MI-inspired relational skills. Considering that reflective learning was an important finding, this aspect could be deepened by exploring the underlying causal mechanisms that lead users to improve their relational skills and to put these latter into practice. Future work could be guided by these research questions: What are the contexts and mechanisms that allow healthcare professionals to integrate MI-consistent relational skills into their professional practice? How does the VP simulation produce different outcomes? Additional research could be conducted to examine how simulation-based intervention change nurses’ practice and how practice-change behaviour translates into patient outcomes (e.g. comfort and quality of life, empowerment, medication adherence).

## Conclusions

Relational skills are fundamental to high-quality nursing care. Findings from this mixed methods study provided critical insight into nurses’ perception of the simulation’s high acceptability. It holds potential to change practice, as nurses become more self-reflective and aware of the impact of their relational skills on patients. VP simulation particularly contributed to knowledge development on MI, on how self-confidence in applying relational skills can be increased by practicing with the VP. Nurses’ participation in the simulation contributed to immersive, positive, and constructive learning experiences. The study highlights the value and novelty of VP simulation for CE in nursing.

## Supplementary Information

**Additional file 1.** CONSORT-EHELTH_UNformat_citations - Reporting guidelines - CONSORT-EHEALTH (Quantitative component of the study).

**Additional file 2.** Questionnaire – Online questionnaire developed for the study.

**Additional file 3.** Pillar integration process – Description of the integration of quantitative and qualitative data and categories.

**Additional file 4.** CHERRIES_UNformat citations - Reporting guidelines – CHERRIES (for the online questionnaire).

**Additional file 5.** COREQ_UNformat citations - Reporting guidelines – COREQ (Qualitative component of the study).

**Additional file 6.** GRAMMS_UNformat citations- Reporting guidelines – GRAMMS (Mixed methods component of the study).

**Additional file 7.** VP simulation design elements – Quantitative findings.

**Additional file 8.** Techno acceptance – Quantitative findings based on the Technology Acceptance Model.

**Additional file 9.** Simulation’ role to support practice – Quantitative findings.

**Additional file 10.** Achievement learning objectives – Quantitative findings.

**Additional file 11.** Operationalization integration – Application of the pillar integration process and an excerpt of the joint display.

## Data Availability

The datasets used and/or analyzed during the current study are available from the corresponding author on reasonable request.

## Abbreviations

CE: Continuing education
CHERRIES: CHEcklist for Reporting Results of Internet E-Surveys
CIHR: Canadian Institutes of Health Research
CONSORT-EHEALTH: Consolidated Standards of Reporting Trials of Electronic and Mobile HEalth Applications and onLine TeleHealth
COREQ: Consolidated Criteria for Reporting Qualitative Studies
GRAMMS: Good Reporting of A Mixed Methods Study
HCP: Healthcare providers
IQR: Interquartile range
INACSL: International Nursing Association for Clinical Simulation and Learning
M: Means
Med: Median
PLHIV: People living with HIV
QUAL: Qualitative
QUANT: Quantitative
MI: Motivational interviewing
SD: Standard deviation
VP: Virtual patient

## References

[1] Denniston C, Molloy E, Nestel D, Woodward-Kron R, Keating JL (2017). Learning outcomes for communication skills across the health professions: a systematic literature review and qualitative synthesis. BMJ Open.

[2] Blackmore A, Kasfiki EV, Purva M (2018). Simulation-based education to improve communication skills: a systematic review and identification of current best practice. BMJ Simulation Technol Enhanced Learning.

[3] Foronda C, MacWilliams B, McArthur E (2016). Interprofessional communication in healthcare: an integrative review. Nurse Educ Pract.

[4] The Joint Commission (2015). Sentinel Event Data Root Causes by Event Type 2004–2014 [http://www.jointcommission.org/assets/1/18/Root_Causes_by_Event_Type_2004-2014.pdf]. Accessed 26 October 2020.

[5] Vermeir P, Vandijck D, Degroote S, Peleman R, Verhaeghe R, Mortier E, Hallaert G, Van Daele S, Buylaert W, Vogelaers D (2015). Communication in healthcare: a narrative review of the literature and practical recommendations. Int J Clin Pract.

[6] Miller WR, Rollnick S (2013). Motivational interviewing: helping people to change.

[7] Rouleau G, Richard L, Côté J, Gagnon M-P, Pelletier J (2019). Nursing practice to support people living with HIV with antiretroviral therapy adherence: a qualitative study. J Assoc Nurses AIDS Care.

[8] Coventry TH, Maslin-Prothero SE, Smith G (2015). Organizational impact of nurse supply and workload on nurses continuing professional development opportunities: an integrative review. J Adv Nurs.

[9] Ellaway R, Candler C, Greene P, Smothers V, & MedBiquitous. (2006). An architectural model for MedBiquitous Virtual Patient [http://groups.medbiq.org/medbiq/display/VPWG/MedBiquitous+Virtual+Patient+Architecture]. Accessed 14 May 2020.

[10] Cant RP, Cooper SJ, Sussex R, Bogossian F (2019). What’s in a name? Clarifying the nomenclature of virtual simulation. Clin Simul Nurs.

[11] Peddle M, Mckenna L, Bearman M, Nestel D (2019). Development of non-technical skills through virtual patients for undergraduate nursing students: an exploratory study. Nurse Educ Today.

[12] Kaplonyi J, Bowles K-A, Nestel D, Kiegaldie D, Maloney S, Haines T, Williams C (2017). Understanding the impact of simulated patients on health care learners’ communication skills: a systematic review. Med Educ.

[13] Bracq M-SM, Michinov E, Jannin P (2019). Virtual reality simulation in nontechnical skills training for healthcare professionals: a systematic review. Simul Healthc.

[14] Cant RP, Cooper SJ (2014). Simulation in the internet age: the place of web-based simulation in nursing education. An integrative review. Nurse Educ Today.

[15] Consorti F, Mancuso R, Nocioni M, Piccolo A (2012). Efficacy of virtual patients in medical education: a meta-analysis of randomized studies. Comput Educ.

[16] Cook DA, Erwin PJ, Triola MM (2010). Computerized virtual patients in health professions education: a systematic review and Meta-analysis. Acad Med.

[17] Kononowicz AA, Woodham LA, Edelbring S, Stathakarou N, Davies D, Saxena N, Car LT, Carlstedt-Duke J, Car J, Zary N (2019). Virtual patient simulations in health professions education: systematic review and Meta-analysis by the digital health education collaboration. J Med Internet Res.

[18] Peddle M, Bearman M, Nestel D (2016). Virtual patients and nontechnical skills in undergraduate health professional education: an integrative review. Clin Simul Nurs.

[19] Cantrell MA, Franklin A, Leighton K, Carlson A (2017). The evidence in simulation-based learning experiences in nursing education and practice: an umbrella review. Clin Simul Nurs.

[20] Rouleau G. [Development and evaluation of a virtual patient simulation aimed to improve nurses’ relational skills in a continuing education context] Développement et évaluation d’une simulation numérique visant à améliorer les habiletés relationnelles des infirmières dans un contexte de formation continue Québec. Canada: Université Laval; 2020.

[21] Sekhon M, Cartwright M, Francis JJ (2017). Acceptability of healthcare interventions: an overview of reviews and development of a theoretical framework. BMC Health Serv Res.

[22] Rouleau G, Pelletier J, Côté J, Gagnon M-P, Martel-Laferrière V, Lévesque R, et al. Codeveloping a virtual patient simulation to foster nurses’ relational skills consistent with motivational interviewing: A situation of antiretroviral therapy nonadherence. J Med Internet Res. 2020;22(7). 10.2196/18225. 10.2196/18225 PMC7391166 32672679

[23] Gottlieb L (2013). Strengths-based nursing care: health and healing for person and family.

[24] Clapper TC (2010). Beyond Knowles: what those conducting simulation need to know about adult learning theory. Clin Simul Nurs.

[25] Eysenbach G, CONSORT-EHEALTH Group (2011). CONSORT-EHEALTH: improving and standardizing evaluation reports of web-based and Mobile health interventions. J Med Internet Res.

[26] Creswell JW, Creswell JD (2018). Research design: qualitative, quantitative, and mixed methods approaches.

[27] Fetters MD, Molina-Azorin J (2020). Utilizing a mixed methods approach for conducting interventional evaluations. J Mix Methods Res.

[28] Johnson RE, Grove AL, Clarke A (2019). Pillar integration process: a joint display technique to integrate data in mixed methods research. J Mix Methods Res.

[29] Pluye P, Kaur N, Granikov V, Garcia BE, Tang D (2018). Mixing phases, results and data in patient oriented research. Int J Multiple Res Approaches.

[30] Campbell C, Stanley J (1963). Experimental and quasi-experimental designs for research.

[31] Deslauriers J-P, Kérisit M, Poupart J, Deslauriers J-P, Groulx L-H, Mayer R, Pirès A (1997). Le devis de recherche qualitative. [Qualitative research design.]. La recherche qualitative: Enjeux épistémologiques et méthodologiques [Qualitative research: Epistemological and methodological issues].

[32] Patton MQ (2015). Qualitative research & evaluation methods.

[33] Le Programme national de mentorat sur le VIH et les hépatites. Le Programme [https://pnmvh.org/a-propos/le-programme/]. Accessed 5 May 2020.

[34] SimforHealth (2020). MedicActiV - Virtual simulation platform for the training of health professionals [http://www.medicactiv.com/en/]. Accessed 23 May 2020.

[35] Cheng Y-M (2012). The effects of information systems quality on nurses’ acceptance of the electronic learning system. J Nurs Res.

[36] Jeffries P (2005). A framework for designing, implementing, and evaluating: simulations used as teaching strategies in nursing. Nurs Educ Perspect.

[37] Fontaine G, Cossette S, Heppell S, Boyer L, Mailhot T, Simard M-J, Tanguay J-F (2016). Evaluation of a web-based E-learning platform for brief motivational interviewing by nurses in cardiovascular care: a pilot study. J Med Internet Res.

[38] Simoneau IL, Paquette C, Fortin F (2012). Traduction et validation en langue française du McCaughey-Traynor Role of Simulation Questionnaire: une étude pilote. (Étude pilote PA2012–015-141EX).

[39] McCaughey CS, Traynor MK (2010). The role of simulation in nurse education. Nurse Educ Today.

[40] Hertzog MA (2008). Considerations in determining sample size for pilot studies. Res Nurs Health.

[41] Thabane L, Ma J, Chu R, Cheng J, Ismaila A, Rios LP, Robson R, Thabane M, Giangregorio L, Goldsmith CH (2010). A tutorial on pilot studies: the what, why and how. BMC Med Res Methodol.

[42] Sidani S, Braden CJ. Testing the Acceptability and Feasibility of Interventions. In: Sidani S, Braden CJ, editors. Design, Evaluation, and Translation of Nursing Interventions. John Wiley & Sons, Ltd; 2011. p. 163–96. 10.1002/9781118785553.ch12.

[43] Kim H-Y (2017). Statistical notes for clinical researchers: chi-squared test and Fisher's exact test. Restor Dent Endod.

[44] Braun V, Clarke V (2006). Using thematic analysis in psychology. Qual Res Psychol.

[45] Paillé P, Mucchielli A (2016). L'analyse qualitative en sciences humaines et sociales.

[46] Cypress BS (2017). Rigor or reliability and validity in qualitative research: perspectives, strategies, reconceptualization, and recommendations. Dimens Crit Care Nurs.

[47] Lincoln YS, Guba EG (1985). Naturalistic inquiry.

[48] Pluye P, Bengoechea EG, Granikov V, Kaur N, Tang DL (2018). A world of possibilities in mixed methods: review of the combinations of strategies used to integrate qualitative and quantitative phases, Results and Data. IJMRA.

[49] Fetters MD, Curry LA, Creswell JW (2013). Achieving Integration in Mixed Methods Designs: Principles and Practices. Health Serv Res.

[50] Fetters MD, Freshwater D (2015). Publishing a methodological mixed methods research article. J Mix Methods Res.

[51] Eysenbach G. Improving the Quality of Web Surveys: The Checklist for Reporting Results of Internet E-Surveys (CHERRIES). J Med Internet Res. 2004;6(3). 10.2196/jmir.6.3.e34. 10.2196/jmir.6.3.e34 PMC1550605 15471760

[52] Tong A, Sainsbury P, Craig J (2007). Consolidated criteria for reporting qualitative research (COREQ): a 32-item checklist for interviews and focus groups. Int J Qual Health Care.

[53] O'Cathain A, Murphy E, Nicholl J (2008). The quality of mixed methods studies in health services research. J Health Serv Res Policy.

[54] Moore J, Donald CK, Sherman L, Pavan M (2018). A conceptual framework for planning and assessing learning in continuing education activities designed for clinicians in one profession and/or clinical teams. Med Teach.

[55] Jeong D, Presseau J, ElChamaa R, Naumann DN, Mascaro C, Luconi F, Smith KM, Kitto S (2018). Barriers and facilitators to self-directed learning in continuing professional development for physicians in Canada: a scoping review. Acad Med.

[56] Billett S (2009). Conceptualizing learning experiences: contributions and mediations of the social, personal, and brute. Mind Cult Act.

[57] International Nursing Association for Clinical Simulation and Learning Standards Committee (2016). Standards of best practice: SimulationSM simulation design. Clin Simul Nurs.

[58] Page-Cutrara K (2014). Use of Prebriefing in nursing simulation: a literature review. J Nurs Educ.

[59] Issenberg SB, Mcgaghie WC, Petrusa ER, Gordon DL, Scalese RJ (2005). Features and uses of high-fidelity medical simulations that lead to effective learning: a BEME systematic review. Med Teach.

[60] Chee J (2014). Clinical simulation using deliberate practice in nursing education: a Wilsonian concept analysis. Nurse Educ Pract.

[61] Bennett-Levy J (2006). Therapist skills: a cognitive model of their acquisition and refinement. Behav Cogn Psychother.

[62] Dieckmann P, Gaba D, Rall M (2007). Deepening the theoretical foundations of patient simulation as social practice. Simul Healthc.

[63] Rudolph JW, Simon R, Raemer DB (2007). Which reality matters? Questions on the path to high engagement in healthcare simulation. Simul Healthc.

[64] Chiniara G, Cole G, Brisbin K, Huffman D, Cragg B, Lamacchia M, Norman D, Canadian Network for Simulation in Healthcare, Guidelines Working Group (2013). Simulation in healthcare: a taxonomy and a conceptual framework for instructional design and media selection. Med Teach.

[65] Moon J (2000). Reflection in learning and professional development: theory and practice.

[66] Gustafsson C, Fagerberg I (2004). Reflection, the way to professional development?. J Clin Nurs.

[67] Tremblay M-C, Richard L, Brousselle A, Beaudet N (2014). Learning reflexively from a health promotion professional development program in Canada. Health Promot Int.

[68] Peddle M (2019). Participant perceptions of virtual simulation to develop non-technical skills in health professionals. J Res Nurs.

[69] Given LM (2008). The sage encyclopedia of qualitative research methods.

[70] Boateng GO, Neilands TB, Frongillo EA, Melgar-Quiñonez HR, Young SL. Best Practices for Developing and Validating Scales for Health, Social, and Behavioral Research: A Primer. Front Public Health. 2018;6(149). 10.3389/fpubh.2018.00149. 10.3389/fpubh.2018.00149 PMC6004510 29942800

