# Supplementary material for: Virtual patient simulation to improve nurses’ relational skills in a continuing education context: a convergent mixed methods study
Source: BMC Nurs. 2022 Jan 4;21:1. doi: 10.1186/s12912-021-00740-x (PMC8725454; doi:10.1186/s12912-021-00740-x)
Supplement: Supplementary file 1 — Additional file 1. CONSORT-EHELTH_UNformat_citations - Reporting guidelines - CONSORT-EHEALTH (Quantitative component of the study). [file 12912_2021_740_MOESM1_ESM.docx]

**Additional file 1. Reporting guidelines: CONSORT-EHEALTH**

CONSORT-EHEALTH: Consolidated Standards of Reporting Trials of Electronic and Mobile HEalth Applications and onLine TeleHealth

We adapted the CONSORT-EHEALTH (1) for reporting information about eHealth and mobile Health trials. CONSORT EHEALTH has been developed for reporting randomized controlled trials. We then adapted the CONSORT E-HEALTH to fit with the pre-experimental design, and we included some statements of the reporting guidelines for Health Care Simulation Research (2).

|  | **Recommendations/**  **statements** | **Page numbers and/or description** |
| --- | --- | --- |
| **Title and abstract** | Indicate the study’s design with a commonly used term in the title or the abstract. | Done. We used “convergent mixed methods study” both in the title and in the abstract. |
|  | Identify the mode of delivery (e.g. web-based, online) in the title. | We used “Virtual patient simulation”. |
|  | Mention primary condition or target group in the title. | We used “Nurses in continuing education” |
|  | Mention key features/functionalities/ components of the intervention in the abstract. If possible, also mention theories and principles used for designing the site. Keep in mind the needs of systematic reviewers and indexers by including important synonyms. | We mentioned this information: “virtual patient simulation to improve nurses’ relational skills in a continuing education context” as well as “consulted the automated virtual patient simulation (informed by motivational interviewing), (p.1). |
|  | In abstract or key terms, the MeSH or searchable keyword term must have the word simulation or simulated. | MeSH: Computer simulation; Simulation training, Virtual patient simulation (p.2) |
| **Introduction** |  |  |
| Background/ rationale | Explain the scientific background and rationale for the investigation being reported. | pp.2-3 |
|  | Clarify whether simulation is the subject of research or the investigational method for research. (SBR) | In our project, VP simulation falls within the category of “subject of research.” According to Cheng et al. (3), simulation as a training methodology examines whether the specific features of simulation contribute to overall educational effectiveness. In our study, we were interested in examining how VP simulation contributed to nurses’ learning experiences, uptake of relational skills, and its transfer to practice. |
| Objectives | State specific objectives, including any prespecified hypotheses | pp.2-3  No hypothesis was formulated. |
| **Methods** |  |  |
| Study design | Present key elements of study design | pp. 3-4 |
|  | A description of changes to methods includes important changes made on the intervention during the trial (e.g. major bug fixes or changes in the functionality or content) and other “unexpected events” that may have influenced study design. | Within the MedicActiv platform, there are two evaluation modes: 1) summative evaluation (or examination mode), with a final “score,” in which learners cannot change their answers during the learning activity; and 2) formative evaluation, which allows learners to change their answers throughout the simulation. The initial evaluation mode was summative evaluation. We discovered this when a participant asked for technical support and then we realized that users could not change their answers. We didn’t want learners to feel as if they were in an “exam period”; rather, we wanted to create a constructive learning experience and low level of stress. We set functionalities so that nurses could change their answers to the quizzes throughout the simulation to allow them to learn from their mistakes (i.e. formative evaluation). This formative evaluation is in line with the clinical team’s vision of continuing professional development. About 50 % had access to one of the other modes of evaluation.  These evaluation modes may have impacted the learners’ experience during the simulation. |
| Setting | Describe the setting, locations, and relevant dates, including periods of recruitment, exposure, follow-up, and data collection | pp. 3-4 Table 1 (p.4). Nurses’ journey in the research process |
|  | Clearly report if outcomes were self-assessed through online questionnaires | pp. 4-5 Yes, the outcomes were self-reported in the online post-test survey. |
| Participants | Give the eligibility criteria, and the sources and methods of selection of participants. | pp. 4-5 |
|  | Specify the recruitment strategies, e.g. open vs. closed, web-based vs. face-to-face assessment. In online-only trials, clarify if participants were quasi-anonymous and whether having multiple identities was possible, and whether technical or logistical measures (e.g. cookies, email confirmation, phone calls) were used to detect/prevent these. | (pp.6-7) The recruitment strategies were hybrid. Online strategies used were: mailing lists (targeting the key population), web banners, and referrals to an open access website with information about the study. Offline strategies consisted of presenting the project during an educational meeting and in nurses’ workplace. Leaflets were distributed. The study was not anonymous as a way of confirming the eligibility criteria (being a nurse) and to make sure that nurses met the requirements for the three-hour accredited CE. Nurses had to provide their name and their email address. Access to the VP simulation was given only when identity of the participant was confirmed. |
| Intervention | Mention names, credentials, and affiliations of the developers, sponsors, and owners (if authors/evaluators are owners or developers of the software, this needs to be declared in a “Conflict of interest” section or mentioned elsewhere in the manuscript). | The competing interests statement contains this information (p.16). |
|  | Describe the theoretical and/or conceptual rationale for the design of each intervention. | Our VP simulation is a narrative approach (4) that depicts a personal story line that progresses over time around the logic of cause and effect, and that involves a decision-making process that yields different “outcomes” or effects. The “good” answers selected by the users are the relational skills that are essential to the proficient practice of MI. These create optimal conditions for relational engagement with patients: asking open-ended questions, using reflective listening, summarizing, affirming the patients’ strengths, providing information and advice, evoking a hypothetical change, eliciting and scaling change talk, setting patient-determined goals, and arriving at a plan. These relational skills are called *behaviour change counselling techniques* because they are active ingredients that allow providers to initiate or maintain communication about health behaviour change (5-7). The “bad answers” are the traps or roadblocks (e.g. expert and blame traps, directive style of counselling) that are inconsistent with MI, and that can cause relational disengagement with the patients. The logic of the VP simulations is as follows: if the nurse uses relational skills consistent with MI, this will open up dialogue with the VP. If the nurse uses relational skills inconsistent with MI, then, the VP will react accordingly (e.g. defensive attitude). Decisions made by users lead to different patient responses. Written standardized feedback is provided at each branching (or decision), in addition to the visualisation of the consequences of these decisions on patient’s speech. The VP simulation has been developed so that users can develop awareness of the impact of relational skills on patient care. |
|  | Describe the history/development process of the application and previous formative evaluations (e.g. focus groups, usability testing), as these will have an impact on adoption/use rates and help with interpreting results | The development process of the VP simulation has been submitted for publication elsewhere (Rouleau et al., 2020)^[[1]](#footnote-1)^ but will be summarized here.  A collaborative and creative approach was used to codevelop the VP simulation. Two main phases were performed: 1) Planning the VP simulation development; and 2) Designing the content, sequence and format of the VP simulation. Phase 1 includes these sub-phases: 1.1) Assessing training needs by understanding HIV nursing practice and its challenges; 1.2) Selecting approaches and theories to inform VP simulation development; 1.3) Negotiating a detailed partnership contract between the research institution, the researcher and the VP simulation company; and 1.4) Assembling an interprofessional team. Phase 2 encompassed these sub-phases: 2.1) Setting the learning objectives and cocreating the clinical content; 2.2) Recording the nurse and patient voice-overs; 2.3) Designing and validating the two-dimensional learning environment; and 2.4) Integrating three modes of fidelity to ensure learner engagement and immersion in the VP simulation.  No previous formative evaluation has been performed. This mixed methods acceptability study is a first step in assessing nurses’ acceptance of this newly developed VP simulation. |
|  | Revisions and updating. Clearly mention the date and/or version number of the intervention evaluated, or describe whether the intervention underwent major changes during the evaluation process, or whether the development and/or content was “frozen” during the study/trial. Describe dynamic components such as news feeds or changing content which may have an impact on the replicability of the intervention | The content of the VP simulation was frozen during the research process. |
|  | Ensure replicability by publishing the source code, and/or providing screenshots/screen-capture video, and/or providing flowcharts of the algorithms used. Replicability is a hallmark of scientific reporting. | A demonstration of the VP simulation is available at: https://www.crsi.umontreal.ca/etudes/simulateur/demo |
|  | Access: Describe how participants accessed the application, in what setting/context, if they had to pay (or were paid) or not, and whether they had to be a member of a specific group. If known, describe how participants obtained “access to the platform and Internet”. To ensure access for editors/reviewers/readers, consider providing a “backdoor” login account or demo mode for reviewers/readers to explore the application. | Once participants filled out the online sociodemographic questionnaire, their identities were verified using their names and email addresses. Then, the student-researcher gave research participants (i.e. nurses) a secured access to the web-based MedicActiv platform (8). Participants were invited to create an individual account to consult the VP simulation, accessible with a computer/laptop or a tablet device with stable Internet connectivity and audio-visual display software. The VP simulation was free of charge and nurses could use the simulation in a convenient location (e.g. workplace, home).  A login account can be provided to editors/reviewers/readers upon request. |
|  | Describe mode of delivery, features/functionalities/components of the intervention and the theoretical framework used to design them. This includes an in-depth description of the content (including where it is coming from and who developed it), “whether [and how] it is tailored to individual circumstances and allows users to track their progress and receive feedback”. This also includes a description of communication delivery channels and – if computer-mediated communication is a component – whether communication was synchronous or asynchronous. | The in-depth description of how MI has been translated into the VP simulation, as well as the composition of the interprofessional team who developed it, are presented elsewhere (Rouleau et al., under revision).  Feedback is standardized, i.e. is provided according to users’ decisions to the various quizzes. Users can get a summary of their decisions and feedback at the end of the VP simulation. This educational intervention is fully automated and represent a set of nurse-patient interactions within a whole consultation, lasting about 45 minutes. |
|  | Describe use parameters (e.g. intended “doses” and optimal timing for use). Clarify what instructions or recommendations were given to the user, e.g. regarding timing, frequency, heaviness of us (if any), or whether the intervention could be used ad libitum. | (p.4, Table 1) During the study, participants were informed that they had unlimited access to the VP simulation. Users could use it *ad libitum* during the research period (March 2019 to August 2019). |
|  | Clarify the level of human involvement (care providers or health professionals, or technical assistance) in the VP simulation. Detail frequency and expertise of professionals involved, if any, as well as “type of assistance offered, the timing and frequency of the support, how it is initiated, and the medium by which the assistance is delivered”. | The student-researcher in charge of the research project was available for technical assistance (email, phone, and videoconference) and for providing additional information about the research process according to users’ need.  The owner of the MedicActiv platform, the company SimForHealth, offered online technical assistance if users clicked on the “Help” button within the VP simulation. To our knowledge, only one nurse used this functionality. |
|  | Report any prompts/reminders used: Clarify if there were prompts (letters, emails, phone calls, SMS) to use the VP simulation, what triggered them, frequency, etc. | E-mails were sent by the student-researcher to nurses at two points in time for different purposes: 1) to invite them to create an account to encourage participants’ uptake, i.e., logins (one initial invitation e-mail was sent and a maximum of three e-mail reminders); 2) to invite them to complete the VP simulation; and 3) to foster their engagement (one initial invitation e-mail was sent and a maximum of three e-mail reminders).  As an incentive the complete the study, three 100$ CAD gift-certificate 300$ CAD total) were raffled off at two points in time. |
|  | Describe any co-interventions (incl. training/support): Clearly state any “interventions that are provided in addition to the targeted eHealth intervention.” | No co-intervention was provided. |
| Outcomes | Define pre-specified outcome measures, including how and when they were assessed. If outcomes were obtained through online questionnaires, describe if they were validated for online use and apply CHERRIES items to describe how the questionnaires were designed/deployed. | pp.4-5  See CHERRIES checklist reporting guidelines (Additional file 2). |
|  | Describe whether and how “use” (including intensity of use/dosage) was defined/measured/monitored (logins, logfile analysis, etc.). Use/adoption metrics are important process outcomes that should be reported in any eHealth trial. | When we planned the project, this engagement measure was used to assess the nurses’ acceptability of the VP simulation: they had to complete 100 % of the intervention.  The “use” was described in terms of completing or not completing the overall VP simulation (“completers” vs “non-completers”). We knew that participants completed the VP simulation because at the end of it, there was a hyperlink that redirected nurses towards the online post-test survey (on LimeSurvey). |
| Data sources/ measurement | For each variable of interest, give sources of data and details of methods of assessment (measurement). | pp. 4-5. For additional information, see CHERRIES checklist reporting guidelines (Additional file 2). |
| Study size | Explain how the study size was arrived at. | p.5 |
| Statistical methods | Describe all statistical methods used. | pp.5 |
| Ethics &  Informed  Consent | Outline informed consent procedures as well as safety and security procedures. | Ethics approval and consent to participate are described at the end of the manuscript. Consent was obtained online by clicking on a checkbox.  Data retrieved from account creation within the VP simulation (i.e., the information needed for account creation, such as name, email address, workplace) were hosted on professional server of Google Compute Platform at Saint-Ghislain, Belgium https://www.google.com/about/datacenters/inside/locations/st-ghislain/  Data gathered from baseline questionnaires and post-test surveys were stored in Canadian server hosted by LimeSurvey, with a privacy policy stated in https://www.limesurvey.org/policies/privacy-policy  All nurses consented to the focus group being recorded. |
| **Results** |  |  |
| Participants | Report numbers of individuals at each stage of study – e.g. numbers potentially eligible, examined for eligibility, confirmed eligible, included in the study, completed follow-up, and analyzed | Figure 4. Flow chart of the completers and non-completers. |
|  | Give reasons for non-participation at each stage |  |
|  | Consider use of a flow diagram |  |
| Baseline descriptive data | In describing characteristics of study participants, include their previous experience with simulation and other demographics associated with digital divide issues, such as age, education, gender, social-economic status, and computer/Internet/eHealth literacy of the participants, if known | Table 3. Nurses’ sociodemographic characteristics, computer literacy skills, MI training and recruitment strategies, pp. 8-9 |
|  | Indicate number of participants with missing data for each variable of interest | There was no missing data because all of the questions were selected as mandatory in LimeSurvey. |
| Recruitment | Dates defining the periods of recruitment and follow-up | Table 1, p.4 |
| Numbers analyzed | Report multiple “denominators” and provide definitions: Report N’s (and effect sizes) “across a range of study participation [and use] thresholds”, e.g., N exposed, N consented, N used more than x times, N used more than y weeks, N participants “used” the intervention at specific pre-defined time points of interest. Always clearly define “use” of the intervention. | This criterion is not fully applicable to our study. We had two groups: 1) completers; 2) non completers. Completers (n=27/49) finished the VP simulation. Among the non-completers, 12 out of 22 created an account in the MedicActiv platform. Only one of these participants (1/12) consulted a part of the VP simulation. |
| Main findings | Presentation of quantitative findings | pp. 4-5, Additional files 7, 8, 9 & 10 |
|  | Presentation of qualitative findings | pp. 9-12 |
|  | Presentation of mixed method interpretations | pp. 12-13 |
| **Discussion** |  |  |
| Key results | Summarize key results with reference to study objectives | p.13 |
| Limitations | Discuss typical limitations of an eHealth trial and limitations of simulation-based research.  Discuss biases due to non-use of the intervention/usability issues, biases through informed consent procedures, and unexpected events. | p.14, and see the GRAMMS checklist (Additional file 6).  Some “non-completers” wrote the student-researcher to inform her of the difficulty in accessing the VP simulation from their workplace and technical issues that prevented them from completing the intervention. |
| Interpretation | Give a cautious overall interpretation of results considering objectives, limitations, multiplicity of analyses, results from similar studies, and other relevant evidence. | The results must be interpreted with caution. Firstly, the sample was highly motivated in participating in the study and had strong computer literacy skills. In general, the participants’ answers were consistently in favor of the VP simulation. This may be explained by the fact that the nurses were highly engaged and motivated to take part in simulation-based research. Secondly, the sample size for the quantitative component was modest, i.e. 27 nurses. See the GRAMMS checklist (Additional file 6). |
| Generalizability | Describe generalizability of simulation-based outcomes to patient-based outcomes (if applicable). | We believe that the simulation-based outcomes are transferable to other French-speaking nurses because the participants’ profiles were varied in terms of sociodemographic characteristics. Another study could be done to assess the extent to which simulation-based training offered to healthcare professionals may influence patients’ outcomes. |
| **Other infor-mation** |  |  |
| Funding | List simulator brand and if conflict of interest for intellectual property exists. | Competing interests and funding are described at the end of the manuscript (p.16). |

VP simulation: Virtual patient simulation; CE: Continuing education; MI: Motivational interviewing

**References**

1. Eysenbach G, CONSORT-EHEALTH Group. CONSORT-EHEALTH: Improving and Standardizing Evaluation Reports of Web-based and Mobile Health Interventions. J Med Internet Res. 2011;13(4):e126.

2. Cheng A, Kessler D, Mackinnon R, Chang TP, Nadkarni VM, Hunt EA, et al. Reporting Guidelines for Health Care Simulation Research: Extensions to the CONSORT and STROBE Statements. Simul Healthc. 2016;11(4):238.

3. Cheng A, Auerbach M, Hunt EA, Chang TP, Pusic M, Nadkarni V, et al. Designing and Conducting Simulation-Based Research. Pediatrics. 2014;133(6):1091-101.

4. Bearman M. Is Virtual the Same as Real? Medical Students’ Experiences of a Virtual Patient. Acad Med. 2003;78(5):8.

5. Dragomir AI, Julien CA, Bacon SL, Boucher VG, Lavoie KL. Training physicians in behavioural change counseling: A systematic review. Patient Educ Couns. 2019;102(1):12-24.

6. Fontaine G, Cossette S, Maheu-Cadotte M-A, Mailhot T, Heppell S, Roussy C, et al. Behavior change counseling training programs for nurses and nursing students: A systematic descriptive review. Nurse Educ Today. 2019;82:37-50.

7. Michie S, Yardley L, West R, Patrick K, Greaves F. Developing and Evaluating Digital Interventions to Promote Behavior Change in Health and Health Care: Recommendations Resulting From an International Workshop. J Med Internet Res. 2017;19(6):e232.

8. SimforHealth. MedicActiV | Virtual simulation platform for the training of health professionals. <http://www.medicactiv.com/en/>. Accessed 18 May 2020.

1. Rouleau G, Pelletier J, Côté J, Gagnon M-P, Martel-Laferrière V, Lévesque R, SimForHealth, Fontaine G: **Codeveloping a virtual patient simulation to foster nurses’ relational skills consistent with motivational interviewing: A situation of antiretroviral therapy nonadherence**. *J Med Internet Res* 2020, **22**(7). [↑](#footnote-ref-1)
