# Supplementary material for: Virtual patient simulation to improve nurses’ relational skills in a continuing education context: a convergent mixed methods study
Source: BMC Nurs. 2022 Jan 4;21:1. doi: 10.1186/s12912-021-00740-x (PMC8725454; doi:10.1186/s12912-021-00740-x)
Supplement: Supplementary file 2 — Additional file 2. Questionnaire – Online questionnaire developed for the study. [file 12912_2021_740_MOESM2_ESM.docx]

Additional file 2. Questionnaire

Hello, nurses.

Thank you for filling out this online questionnaire, which we estimate will take between 15 and 30 minutes of your time.

The following questions are about the virtual patient simulation’s components: the context, glossary, electronic patient record, quizzes, feedback process, labels, and level of fidelity.

You will be asked to rate your degree of disagreement/agreement related to each item.

Your answers are key to helping us understand your impressions and perceptions of using the virtual patient simulation.

Once you have completed the questionnaire, you will have access to your three-hour accredited training session.

| **PART A. Your identification** |
| --- |

At any time, you can click the “Complete Later” button to finish the questionnaire at a later date. You will then have to choose a pseudonym and a password to use when logging into the questionnaire again.

Your first and last names are mandatory for the accredited training in the Faculty of Nursing at the Université de Montréal.

Your e-mail (personal or professional) is also required so we may communicate with you and ensure the follow-up to your participation.

Please present your information in the following format

First name, Last name, E-mail.

| **PART B. Context of the virtual simulation** |
| --- |

The context of the virtual simulation was presented in two formats: by video introduction (12 min.), led by the student researcher, and by text introduction, which included the transcription of the video. Both the video and the text presented background information: the origins of the project, a definition of virtual simulation, its objectives and functioning, the limitations of the approach, and general information on motivational interviewing.

The following questions relate only to these video and text introductions (which have the same content, just different formats).

| Items | Strongly disagree | Disagree | Agree | Strongly agree | N/A  I didn’t consult or watch it |
| --- | --- | --- | --- | --- | --- |
| CONTEXT OF THE VIRTUAL PATIENT SIMULATION/ PREBRIEFING |  |  |  |  |  |
| The student-researcher’s animated video introduction was key to fully understanding the context of the virtual simulation. |  |  |  |  |  |
| The textual introduction (which repeated the same content as the video introduction) was key to fully understanding the context of the virtual simulation. |  |  |  |  |  |
| I think it is important to have access to both information formats (text and video) to understand the context of the virtual simulation. |  |  |  |  |  |

If you have additional comments on the context of the project, you can write it down here:

| **PART C. Glossary** |
| --- |

The glossary refers to the definition and the application of the main terms used in the virtual simulation.

| Items | Strongly disagree | Disagree | Agree | Strongly agree | N/A  I didn’t consult or watch it |
| --- | --- | --- | --- | --- | --- |
| GLOSSARY |  |  |  |  |  |
| The glossary was useful to my learning. |  |  |  |  |  |
| The glossary is a key resource for complementary information about the whole nurse-patient consultation (including quizzes and feedback). |  |  |  |  |  |
| The glossary contained technical terms that were difficult for me to understand. (*negative item*) |  |  |  |  |  |
| I intend to use the glossary as a reference document in the future. |  |  |  |  |  |

Conditional question:

- You indicated that you did not consult the glossary. Would you have consulted it if there had been (click one or more answers): directions that were more visible at the beginning?
- frequent reminders?
- an electronic version of the glossary you would have received by email?
- a hyperlink in each quiz to consult the glossary?
- Other (please provide an answer): _____________________________________________

If you have additional comments on the glossary, you can write it down here:

| **PART D. Electronic patient file** |
| --- |

The patient’s file is made up of the follow sections: the patient’s profile, psychosocial history and vulnerability factors; the patient’s lifestyle, HIV history, and medication history; clinical notes, and the purpose of the consultation.

| Items | Strongly disagree | Disagree | Agree | Strongly agree | N/A  I didn’t consult or watch it |
| --- | --- | --- | --- | --- | --- |
| I received enough information in all the different sections of the “Patient Record” to fully understand the patient’s situation. |  |  |  |  |  |

Conditional questions:

Which section(s) did you feel had insufficient information? Check all that apply and indicate the missing information:

- Patient’s profile

Comment:

- Psychosocial history and vulnerability factors

Comment:

- Lifestyle

Comment:

- HIV history

Comment:

- Medication history

Comment:

- Clinical notes

Comment:

- Purpose of the consultation

Comment:

You have indicated not having consulted the patient’s electronic file. What would have incited you to consult it? Click on one or many answers:

- directions that were more visible at the beginning?
- frequent reminders?
- an electronic version of the patient’s file you would have received by email?
- a hyperlink in each quiz to consult the patient’s electronic file ?
- Other (please provide an answer): _____________________________________________

If you have additional comments on the patient’s file, you can write it down here:

| **PART E. Quizzes, feedback, and labels** |
| --- |

Throughout the interview with the virtual patient, you participated in quizzes. You then received written feedback based on your answers. At certain points in the simulation, labels were shown to you. Illustrated as green and red rectangles, the labels presented keywords to show the nature of the virtual nurse or patient’s interventions. The following questions refer to these quizzes, feedback, and labels.

| Items | Strongly disagree | Disagree | Agree | Strongly agree |
| --- | --- | --- | --- | --- |
| QUIZZES |  |  |  |  |
| The quizzes required I take time to reflect before choosing my answers. |  |  |  |  |
| I saw myself in some of the quiz answers. |  |  |  |  |
| The quizzes made me reflect on my nursing practice. |  |  |  |  |
| There were a sufficient number of quizzes. |  |  |  |  |
| FEEDBACK |  |  |  |  |
| The feedback allowed me to make the connections between the simulated situation and the theoretical elements of MI. |  |  |  |  |
| Feedback was provided in a timely manner (as the consultation progressed). |  |  |  |  |
| Getting the feedback right after the quizzes was disruptive to my learning. |  |  |  |  |
| I would have preferred to get the feedback at the end of the interview. |  |  |  |  |
| I would have liked to have been able to select the format of feedback (audio and/or text). |  |  |  |  |
| VISUAL CUES/LABELS |  |  |  |  |
| I found that the green and red labels next to the dialogue constructively supported my learning. |  |  |  |  |
| Red or green labels were key to qualifying the content of the nurse-patient dialogue. |  |  |  |  |

If you have additional comments on quizzes, feedback and labels, you can write it down here:

| **PART F. Fidelity** |
| --- |

“Fidelity” signifies the virtual simulation’s level of realism, i.e. how much the virtual simulation is like a real-life healthcare situation.

| Items | Strongly disagree | Disagree | Agree | Strongly agree |
| --- | --- | --- | --- | --- |
| The story of the virtual patient who had difficulty following his treatment was realistic. |  |  |  |  |
| The environment in which the interview took place resembled a nurse’s office. |  |  |  |  |
| The virtual patient’s appearance resembled of a typical man living with HIV. |  |  |  |  |
| Virtual simulation realistically reproduced nurse-patient interactions. |  |  |  |  |

If you have additional comments on fidelity, you can write it down here:

| **PART G. The role of simulation in supporting my professional practice** |
| --- |

The following questions relate to how the simulation supported your professional practice in two contexts where medication is taken: 1) among people living with HIV, and 2) among other clienteles.

Rate your disagreement/agreement on a scale ranging from ‘strongly disagree’ to ‘strongly agree’.

Click “not applicable” when the item does not apply to you.

| Items | Strongly disagree | Disagree | Agree | Strongly Agree | N/A  I work exclusively with PLHIV or I don’t work with PLHIV |
| --- | --- | --- | --- | --- | --- |
| The virtual simulation led me to reflect on my nursing practice in the overall healthcare context offered to PLHIV.^a^ |  |  |  |  |  |
| The virtual simulation led me to reflect on my nursing practice as part of antiretroviral treatment support. |  |  |  |  |  |
| Integration of teaching assisted by the virtual simulation will allow me to improve my communication skills with PLHIV. |  |  |  |  |  |
| Integration of teaching assisted by virtual simulation will allow me to improve the health of PLHIV. |  |  |  |  |  |
| Integration of teaching assisted by virtual simulation will allow me to improve the quality of therapeutic relationships with PLHIV. |  |  |  |  |  |
| I feel capable of applying the communication skills seen in the virtual simulation to PLHIV. |  |  |  |  |  |
| My participation in the virtual simulation has made me more confident about facing similar situations with others PLHIV. |  |  |  |  |  |
| The virtual simulation led me to reflect about my nursing practice in general, not just with PLHIV. |  |  |  |  |  |
| Integration of teaching assisted by virtual simulation will lead me to improve my communication skills with clientele other than PLHIV. |  |  |  |  |  |
| Integration of teaching assisted by virtual simulation will allow me to improve the health of other clientele than PLHIV. |  |  |  |  |  |
| Integration of teaching assisted by virtual simulation will lead me to improve the quality of therapeutic relationship with other clientele than PLHIV. |  |  |  |  |  |
| I feel capable of applying the communication skills seen in virtual simulation to other clientele than PLHIV. |  |  |  |  |  |
| My participation in the virtual simulation has made me more confident about facing similar situations with clientele other than PLHIV. |  |  |  |  |  |
| I learned from the mistakes I made in the virtual simulation. |  |  |  |  |  |
| As a result of my virtual simulation, I have identified certain aspects of my professional practice that I could improve. |  |  |  |  |  |
| I learned something new by participating in this virtual simulation. |  |  |  |  |  |
| Integration of teaching assisted by virtual simulation will allow me to increase the use of change talk. |  |  |  |  |  |
| Integration of teaching assisted by virtual simulation will allow me to decrease the use of sustain talk. |  |  |  |  |  |
| The virtual simulation raised my awareness of elements that can facilitate therapeutic relationships with patients. |  |  |  |  |  |
| The virtual simulation has made me aware of the “traps” that can make therapeutic relationships with patients difficult. |  |  |  |  |  |
| My participation in teaching assisted by the virtual simulation has helped me understand how the theoretical notions (from MI^c^) could be applied in my practice. |  |  |  |  |  |
| My participation in teaching assisted by the virtual simulation has been a useful learning experience for my continuing professional development. |  |  |  |  |  |

^a^ PLHIV: people living with HIV

^b^ N/A: not applicable

^c^ MI: motivational interviewing

If you have additional comments on the role of simulation to support nurses’ professional practice, you can write it down here:

| **PART H. Your perspective on the achievement of learning objectives** |
| --- |

Rate your disagreement/agreement as to the extent to which your participation in the virtual simulation allowed you to reach the learning objectives.

| Items | Strongly disagree | Disagree | Agree | Strongly agree | N/A  I already mastered the topic |
| --- | --- | --- | --- | --- | --- |
| Spot traps in nursing interventions that can shut down communication with the patient. |  |  |  |  |  |
| Identify nursing interventions that optimize openness to the patient’s experience. |  |  |  |  |  |
| Apply nursing interventions that elicit change talk. |  |  |  |  |  |
| Spot traps in nursing interventions that enable the statu quo (sustain talk). |  |  |  |  |  |
| Identify cues in the patient’s speech that reflect change talk. |  |  |  |  |  |
| Target the key elements that are important to include in providing information to the patient. |  |  |  |  |  |
| Identify principles to build an action plan with the patient. |  |  |  |  |  |
| Describe the principles consistent with MI that structure information sharing with the patient. |  |  |  |  |  |

^a^ N/A: Not applicable means, “I already mastered the topic.”.

Did your participation in the virtual simulation allow you to achieve other learning objectives that have not been previously mentioned?

- Yes (please specify)
- No

If you have additional comments on the learning objectives, write them here:

| **PART I: Acceptability questionnaire** |
| --- |

The following questions focus on your appreciation of the virtual simulation.

These questions are not intended to verify your knowledge. There are no bad or good answers. On a scale ranging from ‘strongly disagree’ to ‘strongly agree,’ indicate your disagreement/agreement with each item.

| Items | Strongly disagree | Disagree | Agree | Strongly  agree | N/A |
| --- | --- | --- | --- | --- | --- |
| **System quality** |  |  |  |  |  |
| The virtual simulation can gave learners control over their learning activity. |  |  |  |  |  |
| The virtual simulation presented course materials in a multimedia and readable format. |  |  |  |  |  |
| The virtual simulation can offer flexibility in learning as to time. |  |  |  |  |  |
| The virtual simulation can offer flexibility in learning as to place. |  |  |  |  |  |
| The digital simulation was interactive. |  |  |  |  |  |
| **Information quality** |  |  |  |  |  |
| The content of the virtual simulation was innovative. |  |  |  |  |  |
| The virtual simulation met my learning needs. |  |  |  |  |  |
| The level of difficulty of the virtual simulation learning content was appropriate. |  |  |  |  |  |
| **Service quality** |  |  |  |  |  |
| I acquired adequate support within the virtual simulation to help my learning (e.g. user guide). |  |  |  |  |  |
| I acquired adequate support from the virtual simulation’s administrators for the app’s technical aspects. |  |  |  |  |  |
| Overall, support services of the virtual simulation were satisfactory. |  |  |  |  |  |
| **User-interface design quality** |  |  |  |  |  |
| The layout of the virtual simulation was user friendly. |  |  |  |  |  |
| The layout of the virtual simulation was well structured. |  |  |  |  |  |
| Overall, user-interface design of the virtual simulation was satisfactory (e.g. overall design, 3D images, colours, consultation processes). |  |  |  |  |  |
| **Perceived usefulness** |  |  |  |  |  |
| Using the virtual simulation seemed to me to be more effective than other types of training I might have received. |  |  |  |  |  |
| Using the virtual simulation enhanced the effectiveness of my learning. |  |  |  |  |  |
| I found the virtual simulation to be useful in my learning. |  |  |  |  |  |
| **Perceived ease of use** |  |  |  |  |  |
| Using virtual simulation did not require a lot of mental effort. |  |  |  |  |  |
| I have found the virtual simulation to be easy to use. |  |  |  |  |  |
| I quickly developed ease in using the virtual simulation. |  |  |  |  |  |
| **Perceived enjoyment** |  |  |  |  |  |
| I have found using the virtual simulation enjoyable. |  |  |  |  |  |
| The navigation within the virtual simulation was pleasant. |  |  |  |  |  |
| I have had fun using the virtual simulation. |  |  |  |  |  |
| **Intention to use** |  |  |  |  |  |
| I would use the virtual simulation on a regular basis in the future, if it were available online. |  |  |  |  |  |
| I would use the virtual simulation frequently in my practice, if it were available online. |  |  |  |  |  |
| I would strongly recommend the virtual simulation be made available to other nurses. |  |  |  |  |  |
| I would strongly recommend the virtual simulation be made available to other healthcare professionals. |  |  |  |  |  |

| **PART J. Open-ended questions** |
| --- |

What were the elements that most caught your interest?

Which elements did you like the least?

What recommendations would you make with a view to improving the virtual simulation?

Do you have anything else to add (optional)?

| **PART K. Your advice on the questionnaire** |
| --- |

Throughout the questionnaire, the answers scales were formulated as: strongly disagree, disagree, agree and strongly agree:

There was no choice of a neutral value (i.e. neither disagree nor agree).

Please indicate, pour each statement, the answer that best reflects how you feel:

| Items |  |  |  |  |  |
| --- | --- | --- | --- | --- | --- |
| Throughout the questionnaire, selecting an answer without being able to position myself “neutrally” (neither disagreeing nor agreeing) was: | Very difficult | Difficult | Neither difficult nor easy | Easy | Very easy |
| Throughout the questionnaire, if I had had the opportunity to choose a neutral option (neither in disagree nor agree), this position would have: | Definitely changed my answers. | Slightly changed my answers. | Made no difference to my answers. |  |  |
| Since I did not have the opportunity to choose a neutral option when filling out the questionnaire, I felt: | Completely forced to take a position. | Forced to take a position. | Neither forced nor free to take a position. (I was indifferent and didn’t think about it.) | Free to take a position. | Completely free to take a posi-tion. |
