# Supplementary material for: Virtual patient simulation to improve nurses’ relational skills in a continuing education context: a convergent mixed methods study
Source: BMC Nurs. 2022 Jan 4;21:1. doi: 10.1186/s12912-021-00740-x (PMC8725454; doi:10.1186/s12912-021-00740-x)
Supplement: Supplementary file 3 — Additional file 3. Pillar integration process – Description of the integration of quantitative and qualitative data and categories. [file 12912_2021_740_MOESM3_ESM.docx]

Additional file 3. Description of the pillar integration process

We used the four stages of the pillar integration process as the strategy for integrating the quantitative and qualitative findings (1): 1) Listing; 2) Matching; 3) Checking; 4) Pillar building.

*Stage 1 – Listing:* This involves selecting the relevant raw quantitative data that can be listed in the column (QUANT data), i.e. items in the survey along with their description statistics – means and standard deviations, as well as grouped data in the QUANT categories (e.g. variables). One example of a raw quantitative data was: “The story of the virtual patient who had difficulty following his treatment was realistic (3.56±0.58)”. This data belongs to the “fidelity” quantitative category. Given the huge numbers of items and data in the quantitative survey (n=80 items), we prioritized the listing of quantitative data that can be compared to the qualitative data (e.g., features of the VP simulation, roles/impact of the VP simulation).

*Stage 2 – Matching:* QUAL data (verbatim) and categories (themes) are then listed in their respective columns to match the QUANT data and categories columns. The organization and comparison of the data and categories can identify patterns, similarities or differences. If no match is found between QUANT and QUAL, a mention “not identified” can be written. Given the huge numbers of items and data in the quantitative survey (n=80 items), we prioritized the listing of quantitative data that can be compared to the qualitative data (e.g., features of the VP simulation, roles/impact of the VP simulation). One QUAL data that matched the example provided in Stage 1 was: “*The patient’s story was really, um, it’s real life, it’s really believable; it’s not something just pulled out of thin air*.” This verbatim supported the QUAL category (theme) as follows: Enjoying the practice in a realistic, immersive and non-judgmental environment.

*Stage 3 – Checking:* This stage is about cross-checking the QUANT and QUAL data and their emerging patterns (or lack thereof), for quality-integration purposes. An iterative process makes it possible to go back to the data and look for the accuracy, appropriateness and completeness of the matches (or absence of matches).

*Stage 4 – Pillar building:* Here*,* the QUANT and QUAL findings are compared and contrasted from the listing, matching, and checking phases. Insights are conceptualized from the connection of the QUANT and QUAL columns. Both types of data and categories are integrated and connected to build inferences on patterns, themes, or insights, and possible explanations. When the pillar is finalized, the researcher can write a meaningful narrative to relate mixed evidence. This is what we did by using a weaving approach (2, 3) and grouping quantitative and qualitative findings under mixed method interpretations. Based on the examples provided in Stage 1 and Stage 2, we ended up with this mixed method interpretation finding: Influence of the simulation’s fidelity on nurses’ impression of getting real practice and of having an immersive learning experience. The narrative combining the integration of the quantitative and qualitative findings meant to answer the third mixed methods research objective around understanding how the VP simulation contributed to nurses’ uptake of relational skills, to overall learning and its transfer into practice.

**References**

1. Johnson RE, Grove AL, Clarke A. Pillar Integration Process: A Joint Display Technique to Integrate Data in Mixed Methods Research. J Mix Methods Res. 2019;13(3):301-20.

2. Fetters MD, Curry LA, Creswell JW. Achieving Integration in Mixed Methods Designs: Principles and Practices. Health Serv Res. 2013;48(6pt2):2134.

3. Fetters MD, Freshwater D. Publishing a Methodological Mixed Methods Research Article. J Mix Methods Res. 2015;9(3):203-13.
