# Supplementary material for: Virtual patient simulation to improve nurses’ relational skills in a continuing education context: a convergent mixed methods study
Source: BMC Nurs. 2022 Jan 4;21:1. doi: 10.1186/s12912-021-00740-x (PMC8725454; doi:10.1186/s12912-021-00740-x)
Supplement: Supplementary file 4 — Additional file 4. CHERRIES_UNformat citations - Reporting guidelines – CHERRIES (for the online questionnaire). [file 12912_2021_740_MOESM4_ESM.docx]

**Additional file 4. CHERRIES: CHEcklist for Reporting Results of Internet E-Surveys**

We used this reporting guideline (1) regarding the data collection method used (online post-test survey) for the quantitative component.

| **Item Category** | **Checklist Item** | **Explanation** | **Strategies used in our VP simulation-based research** |
| --- | --- | --- | --- |
| **Design** |  |  |  |
|  | Describe survey design | Describe target population, sample frame. Is the sample a convenience sample? (In “open” surveys this is most likely.) | We used a combination of convenience and snowballing sampling strategies. We wanted a sample of nurses that were interested in and available to spend time participating in the simulation-based research. p.4 |
| **IRB (Institutional Review Board) approval and informed consent process** | | | |
|  | IRB approval | Mention whether the study has been approved by an IRB. | Yes- approval from University of Montreal Hospital Center IRB [#18.243]. |
|  | Informed consent | Describe the informed consent process. Were the participants told the length of time of the survey, which data were stored and where and for how long, who the investigator was, and the purpose of the study? | Participants had online access to the information and consent form, and they could have downloaded an electronic copy.  All of the following information was presented: the principal investigators, co-investigators and collaborators; purpose of the study; number of study participants and length of the participation; nature of the participation requested; conduct of the study/procedures; risks and inconveniences; benefits; confidentiality; communication of overall results; funding of the project; compensation; voluntary participation and the right to withdraw; and identification of contact people.  Information about which specific data related to their registration on the MedicActiv platform as well as the data retrieved from the baseline questionnaire and post-intervention survey was provided (LimeSurvey), along with the servers that host/store these data. Two URL were provided for further information of privacy policies.  After this, participants had to consent by clicking “approve” at the bottom of the consent screen. Then, they had to click on whether or not they possessed a valid nurse’s practice license. If they answered “yes”, they were directed to the baseline or pre-intervention questionnaire that contains these information: sociodemographic, computer literacy skills, MI training, recruitment strategies. |
|  | Data protection | If any personal information was collected or stored, describe what mechanisms were used to protect unauthorized access. | The sociodemographic questionnaire and the post-test survey were not anonymous, given our need to know participants’ identities to be able to assign them their CE credits. Identifiable information (complete name and e-mail address) was collected in order to verify the identity of nurses and to check their registration to the VP simulation platform.  The data were stored on a secure database on the LimeSurvey server (for the online baseline questionnaire and post-test survey) and on the MedicActiv server. |
| **Development and pre-testing** | | | |
|  | Development and testing | State how the survey was developed, including whether the usability and technical functionality of the electronic questionnaire had been tested before fielding the questionnaire. | Two rounds of survey pre-tests were conducted before the survey. The first was on paper and/or electronic versions, and was done by seven people with expertise in research, nursing care, and/or learning program development and evaluation. Participants’ comments in the first round led us to clarify some statements, improve the visual presentation, divide statements containing multiple ideas, and withdraw the midpoint response. Afterwards, five potential end users and one research-coordinator completed the online questionnaire on LimeSurvey. They found the navigation to be user-friendly and deemed the visual interface to be suitable. Their comments led us to clarify the content of certain statements.  We removed the midpoint response from the Likert-scale (neither disagree nor agree) based on suggestions of experts in program evaluation that participated in the survey pre-test and in the literature (2). The midpoint response may have various interpretations; possible neutral responses were either “no opinion” or “I don’t know”. These midpoint responses provided very little information (for example, identifying areas for future improvement of the simulation). |
| **Recruitment process and description of the sample having access to the questionnaire** | | | |
|  | Open survey versus closed survey | An “open survey” is a survey open for each visitor of a site, while a closed survey is only open to a sample which the investigator knows (password-protected survey). | Information about the study was sent out to target groups. The URL to access the baseline questionnaire was available once participants consented to take part in the study and met the eligibility criteria. The access to the VP simulation platform was closed and protected by a unique URL that was sent manually by the student-researcher after participant’s identity was confirmed. |
|  | Contact mode | Indicate whether or not the initial contact with the potential participants was made on the Internet. (Investigators may also send out questionnaires by mail and allow for Web-based data entry.) | The initial contacts were either by email or in-person. |
|  | Advertising the survey | How/where was the survey announced or advertised? Some examples are offline media (newspapers), or online (mailing lists – If yes, which ones?) or banner ads (Where were these banner ads posted and what did they look like?). It is important to know the wording of the announcement as it will heavily influence who chooses to participate. Ideally the survey announcement should be published as an appendix. | The study was advertised online and offline (in person).  Study information was sent through mailing lists and newsletters with banners ads, all sent by the HIV mentoring national program and by the Order of Nurses of Quebec.  Text of the banner ads: “Better care through better communication: three hours of accredited online training. Sign up.” |
| **Survey administration** | |  |  |
|  | Web/E-mail | State the type of e-survey (e.g. one posted on a Web site, or one sent out through e-mail). If it is an e-mail survey, were the responses entered manually into a database, or was there an automatic method for capturing responses? | The URL that provided access to the study was sent out through e-mail, and was available by clicking on the banner ads as well as on the leaflet.  The web-based data entry was an automatic method used by the LimeSurvey platform. |
|  | Context | Describe the Website (for mailing list/newsgroup) in which the survey was posted. What is the Website about, who is visiting it, what are visitors normally looking for? Discuss to what degree the content of the Website could pre-select the sample or influence the results. For example, a survey about vaccination on an anti-immunization Website will have different results from a Web survey  conducted on a government Website | Participants receiving study information through e-mail could access the study directly. If participants clicked on the banner ads, they were directed to a page within the website of the Research Chair in Innovative Nursing Practices ([www.crsi.umontreal.ca](http://www.crsi.umontreal.ca)). This Research Chair is grounded in a high-quality research program in nursing and in interdisciplinary fields of research; this is a credible website. |
|  | Mandatory/voluntary | Was it a mandatory survey to be filled in by every visitor who wanted to enter the Website, or was it a voluntary survey? | Participants could choose to take part in the study or not. The post-test survey was mandatory if participants wanted to obtain their CE credits. |
|  | Incentives | Were any incentives offered (e.g. monetary, prizes, or non-monetary incentives such as an offer to provide the survey results)? | Participants gained three hours of accredited CE for completing the post-test survey and the VP simulation. They were also entered into prize draws (three 100$ gift-certificates). |
|  | Time/Date | In what timeframe were the data collected? | The data were collected for the baseline questionnaire, for account registration into the VP simulation platform, and for the baseline questionnaire, without any specified interval over a five month period. |
|  | Randomization of items or questionnaires | To prevent biases items can be randomized or alternated. | The questions were not randomized. |
|  | Adaptive questioning | Use adaptive questioning (certain items, or only conditionally displayed based on responses to other items) to reduce number and complexity of the questions. | Adaptive questioning was used. |
|  | Number of Items | What was the number of questionnaire items per page? The number of items is an important factor for the completion rate. | The baseline questionnaire included 14 questions.  The number of items per page varied depending on the structure of the post-test survey. A total of 80 items had to be completed. |
|  | Number of screens (pages) | Over how many pages was the questionnaire distributed? The number of items is an important factor for the completion rate. | This information is no longer available. |
|  | Completeness check | It is technically possible to do consistency or completeness checks before the questionnaire is submitted. Was this done, and if “yes”, how (usually JAVAScript)? An alternative is to check for completeness after the questionnaire has been submitted (and highlight mandatory items). If this has been done, it should be reported. All items should provide a non-response option such as “not applicable” or “rather not say”, and selection of one response option should be enforced. | All questions had to be completed. We used non-response options “not applicable”, or “I prefer to not answer” for both the baseline questionnaire and the post-test survey. |
|  | Review step | State whether respondents were able to review and change their answers (e.g. through a Back button or a Review step which displays a summary of the responses and asks the respondents if they are correct). | Respondents were able to go back and review questions. |
| **Response rates** | |  | |
|  | Unique site visitor | If you provide view rates or participation rates, you need to define how you determined a unique visitor. There are different techniques available, based on IP addresses or cookies or both. | N/A |
|  | View rate (Ratio of unique survey visitors/unique site visitors) | Requires counting unique visitors to the first page of the survey, divided by the number of unique site visitors (not page views!). It is not unusual to have view rates of less than 0.1 % if the survey is voluntary. | N/A |
|  | Participation rate (Ratio of unique visitors who agreed to participate/unique first survey page visitors) | Count the unique number of people who filled in the first survey page (or agreed to participate, for example by checking a checkbox), divided by visitors who visit the first page of the survey (or the informed consents page, if present). This can also be called “recruitment” rate. | This information is not available. |
|  | Completion rate (Ratio of users who finished the survey/users who agreed to participate) | The number of people submitting the last questionnaire page, divided by the number of people who agreed to participate (or submitted the first survey page). This is only relevant if there is a separate “informed consent” page or if the survey goes over several pages. This is a measure for attrition. Note that “completion” can involve leaving questionnaire items blank. This is not a measure for how completely questionnaires were filled in. (If you need a measure for this, use the word “completeness rate”.) | Of the 54 participants that started to fill out the baseline questionnaire, 49 completed it. From that point, 27 completed their overall participation into the web-based study (i.e. completed the VP simulation as well as the post-intervention questionnaire). See Figure 4. Flow chart of the completers and non-completers. |
| **Preventing multiple entries from the same individual** | | | |
|  | Cookies used | Indicate whether cookies were used to assign a unique user identifier to each client computer. If so, mention the page on which the cookie was set and read, and how long the cookie was valid. Were duplicate entries avoided by preventing users’ access to the survey twice; or were duplicate database entries having the same user ID eliminated before analysis? In the latter case, which entries were kept for analysis (e.g. the first entry or the most recent)? | No cookies were used. The student-researcher carefully checked for duplicate participants, because they provided their name and e-mail address. Two participants completed the study twice. We considered only their first entry. |
|  | IP check | Indicate whether the IP address of the client computer was used to identify potential duplicate entries from the same user. | N/A |
|  | Log file analysis | Indicate whether other techniques to analyze the log file for identification of multiple entries were used. If so, please describe. | N/A |
|  | Registration | In “closed” (non-open) surveys, users need to login first and it is easier to prevent duplicate entries from the same user. Describe how this was done. | N/A |
| **Analysis** | | | |
|  | Handling of incomplete questionnaires | Were only completed questionnaires analyzed? Were questionnaires which terminated early (where, for example, users did not go through all questionnaire pages) also analyzed? | We analyzed only completed questionnaires. |
|  | Questionnaires submitted with an atypical timestamp | Some investigators may measure the time people needed to fill in a questionnaire and exclude questionnaires that were submitted too soon. Specify the timeframe that was used as a cut-off point, and describe how this point was determined. | N/A |
|  | Statistical correction | Indicate whether any methods such as weighting of items or propensity scores have been used to adjust for the non- representative sample; if so, please describe the methods. | N/A |

CE: continuing education; N/A: not applicable; VP simulation: virtual patient simulation

**References**

1. Eysenbach G. Improving the Quality of Web Surveys: The Checklist for Reporting Results of Internet E-Surveys (CHERRIES). J Med Internet Res. 2004;6(3).

2. Raaijmakers QAW. Adolescents' midpoint responses on Likert-type scale items: Neutral of missing values ? Int J Public Opin Res. 2000;12(2):209-17.
