# Supplementary material for: Virtual patient simulation to improve nurses’ relational skills in a continuing education context: a convergent mixed methods study
Source: BMC Nurs. 2022 Jan 4;21:1. doi: 10.1186/s12912-021-00740-x (PMC8725454; doi:10.1186/s12912-021-00740-x)
Supplement: Supplementary file 5 — Additional file 5. COREQ_UNformat citations - Reporting guidelines – COREQ (Qualitative component of the study). [file 12912_2021_740_MOESM5_ESM.docx]

**Additional file 5. COREQ: Consolidated Criteria for Reporting Qualitative Studies**

We used this reporting guideline for the qualitative component (1).

| No. | Item | Description |
| --- | --- | --- |
| **Domain 1: Research team and reflexivity** | | |
| Personal characteristics | | |
| 1. | Interviewer/facilitator | The Student-Research, GR |
| 2. | Credentials | GR – Student-Researcher – MSc, PhD candidate (Nursing) |
|  |  | MPG – PhD (Public Health) |
|  |  | JC – PhD (Nursing) |
|  |  | LR – BSc (Hons), PhD (Nursing) |
|  |  | GC – MSc, PhD candidate (Nursing) |
|  |  | JP – MSc, PhD candidate (Nursing) |
| 3. | Occupation | GR – RN, PhD candidate in nursing (Université Laval, Canada); Research Chair coordinator (University of Montreal Hospital Research Centre, Canada). |
|  |  | MPG – Full Professor (Nursing Faculty, Université Laval, Canada), Researcher and Chair Holder (Research Centre in Quebec City, Canada) |
|  |  | JC – RN, Full Professor (Nursing Faculty, University of Montreal, Canada); Researcher and Chair Holder (University of Montreal Hospital Research Centre, Canada) |
|  |  | LR – RN, Research Fellow (University of Otago, New Zealand); Associate Professor (Nursing Faculty, University of Montreal, Canada); Hon. Research Fellow (University of Melbourne) |
|  |  | GC – RN, PhD candidate (Nursing Faculty, University of Montreal); Research and clinical advisor (University of Montreal Hospital Centre) |
|  |  | JP – Professor (Université du Québec à Rimouski, Canada); PhD candidate in nursing (Université Laval) |
| 4. | Gender | Five female-identifying and one male-identifying researchers (interviewer: female-identifying). |
| 5. | Experience and training | GR –has experience in developing and evaluating virtual nursing interventions in antiretroviral therapy (ART) adherence, in conducting qualitative research, and in knowledge synthesis. |
|  |  | MPG – has expertise in implementation science, mixed methods methodologies, knowledge-transfer, evidence-based decision-making, and evaluation of information and communication technologies (ICTs). |
|  |  | JC – has expertise in developing and evaluating nursing and no-nursing interventions among people living chronic conditions, such as people living with HIV (PLHIV), in the context of ART adherence. |
|  |  | LR – has expertise in qualitative research and methodologies, including refugee health services research, and access to primary health care for vulnerable populations. |
|  |  | GC – has clinical expertise with clientele having concurrent disorders, and has interests in mixed methods methodologies and in nurses’ continuing education. |
|  |  | JP – has clinical experience with PLHIV, is interested by HIV-related stigma, and has experience in using motivational interviewing. |
| Relationship with participants | | |
| 6. | Relationship established | GR animated the focus group. She knew the majority of participants prior to this study. GR was directly involved in participant recruitment, data collection and analysis. She encouraged participants to express themselves freely, by sharing their *true* impressions of the VP simulation and not what they thought the student-researcher wanted to hear (in order to reduce the desirability bias). Knowing about the elements they least appreciated can help improve the VP simulation. |
| 7. | Participant knowledge of interviewer | GR introduced herself to participants as a PhD nursing student at Université Laval who leads the simulation-based research. She also introduced the research team. |
| 8. | Interviewer characteristics | RN, PhD candidate, simulation-based research leader and principal investigator, under the supervision of MPG and JC. |
| **Domain 2: Study design** | | |
| Theoretical framework | | |
| 9. | Methodological orientation and theory | We used a qualitative exploratory design to take into account nurses' description of their simulation-based learning experience. We performed a thematic analysis. |
| Participant selection | | |
| 10. | Sampling | p.4 |
| 11. | Method of approach | Twelve nurses out of 27 that completed the VP simulation and the post-test survey didn’t accept to be re-contacted for the qualitative component. The remaining 15 participants were contacted by email to invite them to take part in the qualitative component. |
| 12. | Sample size | Five nurses participated in the focus group. |
| 13. | Non-participation | N/A |
| Setting | | |
| 14. | Setting of data collection | p. 5 Zoom videoconferencing platform. |
| 15. | Presence of non-participants | N/A |
| 16. | Description of sample | Table 3 (pp.8-9). Nurses’ sociodemographic characteristics, computer literacy skills, MI training and recruitment strategies. |
| Data collection | | |
| 17. | Interview guide | Some examples from the focus group topic guide are presented in Table 2 (p.6).  GR sent the guide to the five nurses prior to the focus group, allowing them to look at the questions. |
| 18. | Repeat focus group | No |
| 19. | Audio/visual recording | Audio and visual recording (with Zoom) |
| 20. | Field notes | Field notes were taken during the focus group by a research coordinator who was a “non-participant observer”. |
| 21. | Duration | The focus group lasted 75 minutes. |
| 22. | Data saturation | We achieved an inductive and a priori thematic saturation (2). The first type of saturation, inductive saturation, means that new themes/topics emerged from those initially identified in the focus group guide (e.g. the difference between learning MI through VP simulation and face-to-face). The second type, a priori saturation, relates to the degree to which identified themes/topics covered in the focus group guide are exemplified by each participant. |
| 23. | Transcripts returned | No participant received the focus group transcript. |
| **Domain 3: Analysis and findings** | | |
| Data analysis | | |
| 24. | Number of data coders | Coding was led by GR and involved comparison across transcripts. |
| 25. | Description of the coding tree | We didn’t describe the coding tree, but we clearly defined the themes. |
| 26. | Derivation of themes | GR assessed the descriptive value of the themes against the transcripts. The other team members were involved in discussions of preliminary thematic findings and in the qualitative and mixed methods interpretation findings. |
| 27. | Software | NVivo Pro version 12 |
| 28. | Participant checking | N/A |
| Reporting | | |
| 29. | Quotations presented | Illustrative quotes support the presentation of findings while participants' anonymity was respected (pp.9-12). |
| 30. | Data and findings consistent | The findings are strongly supported by the qualitative data. |
| 31. | Clarity of major themes | Major themes are clearly identified. |
| 32. | Clarity of minor themes | Subthemes are clearly identified and related to major themes. |
| ART: antiretroviral therapy; I: Investigator; ICTs: information and communication technologies; N/A: not applicable; PLWH: people living with HIV; RN: registered nurse  **References** | | |

1. Tong A, Sainsbury P, Craig J. Consolidated criteria for reporting qualitative research (COREQ): A 32-item checklist for interviews and focus groups. Int J Qual Health Care. 2007;19(6):349-57.

2. Saunders B, Sim J, Kingstone T, Baker S, Waterfield J, Bartlam B, et al. Saturation in qualitative research: exploring its conceptualization and operationalization. Qual Quant. 2018;52(4):1893-907.
