# Supplementary material for: Virtual patient simulation to improve nurses’ relational skills in a continuing education context: a convergent mixed methods study
Source: BMC Nurs. 2022 Jan 4;21:1. doi: 10.1186/s12912-021-00740-x (PMC8725454; doi:10.1186/s12912-021-00740-x)
Supplement: Supplementary file 6 — Additional file 6. GRAMMS_UNformat citations- Reporting guidelines – GRAMMS (Mixed methods component of the study). [file 12912_2021_740_MOESM6_ESM.docx]

**Additional file 6. GRAMMS: Good Reporting of A Mixed Methods Study**

We added complementary information in the GRAMMS (1) to support the rationale behind our choice of a mixed methods study.

| **Criteria** | **Complementary information and reference to the manuscript** |
| --- | --- |
| 1. Describe the justification for using a mixed methods approach to the research question. | There is a paucity of evidence regarding the use of VP simulation to support nurses’ continuing education. We developed a novel VP simulation with a view to improving nurses’ relational skills. It is recommended to assess the acceptability of an intervention (2) before planning a larger evaluative study. By combining two complementary types of methods, data and results, the mixed methods approach was therefore an appropriate research type for gathering richer descriptions and a broader understanding nurses’ perception of the VP’s acceptability and their learning experiences during this innovative training technique. The main rationale for a mixed methods study was based on its completeness (3), and complementarity: we sought a comprehensive account of nurses’ perceptions of the VP’s acceptability, and a greater understanding of how VP simulation can contribute to their learning progression and transfer of such learning into practice (mixed methods integration research question). |
| 1. Describe the design in terms of the purpose, priority, and sequence of methods. | Convergent mixed methods design occurs when both quantitative and qualitative data are collected and analyzed in the same phase of the research process. The two sets of results are then merged to produce an overall interpretation. Quantitative and qualitative methods have an equal priority and both play an important role in exploring the research question (4). Quantitative and qualitative components were interdependent (5) due to the concurrent timing of the methods, the engagement of the same student-researcher throughout the research process, and the research participants. In our case, we collected and analyzed quantitative data first because nurses had to complete the VP simulation and post-test survey before taking part in the qualitative data collection. The timing of the methods was concurrent because both were part of a single research phase. The student-researcher was involved in both the quantitative and qualitative strands. She was not blinded from the quantitative data and findings when she undertook the qualitative component. The other interdependence was that the same group of participants was involved in both strands. The qualitative sample was drawn from the quantitative component. The data are therefore interdependent. |
| 1. Describe each method in terms of sampling, data collection, and analysis. | Convergent mixed methods study: pp. 3-4  Quantitative component: pp.4-5  Qualitative component : pp. 5-6 |
| 1. Describe where integration has occurred, how it has occurred, and who has participated. | We aimed to merge the quantitative and qualitative results in order to understand how the quality and design elements of the VP simulation contributed to nurses’ learning experience, their acquisition or consolidation of relational skills, and the transfer of these latter into practice. Integration occurred when both quantitative and qualitative data were collected and analyzed. We used a comparison-of-results strategy using the pillar process technique (6) and a joint display (see Additional files 3 and 11). The student-researcher led the overall integration, supported by the co-authors’ input. Two of the co-authors were skilled in mixed methods studies. |
| 1. Describe any limitation of one method associated with the present of the other method. | The mixed methods study approach has strengths and weaknesses pertaining to quantitative research, qualitative research, and mixed research (7). In other words, the strengths of the two methods are cumulative, as are their limitations (8). An important limitation related to the quantitative component was the lack of conceptual clarity regarding the items covered in the overall questionnaire. With the exception of the Global System Quality and Technology Acceptance -known as Technology Acceptance Model (9), the survey was not validated. For example, in the *role of simulation to support nurses’ practice* tool, many items could have been conceptualized and operationalized around distinct variables (e.g. self-efficacy, perceived effectiveness). The mixed methods design is a strength because the interviews covered aspects that were little or not at all covered in the measurement tools. This helped to refine our understanding of the nurses’ learning experience. In the qualitative component, the five-participant sample size was relatively small. The student-researcher led this focus group. She knew the quantitative results, and led the overall mixed methods study. Although she was aware of her potential personal biases, she may nonetheless have unconsciously influenced the research process. However, the co-authors, including the thesis supervisors, were involved in the entire research process. Two main challenges were encountered during the integration. The first was dealing with huge numbers of items and data in the quantitative survey (n=80 items) and comparing these to the qualitative data that was focused on circumscribed thematic. The second was the unequal sample size (27 in the quantitative component; and 5 in the qualitative part). Use of the joint display was helpful in putting together data and results that can be compared and discussed. Furthermore, collecting quantitative and qualitative data from the same participants may have mitigated the limitations of the non-validated questionnaire. Contradictions between quantitative and qualitative results is a challenge or a limitation of the convergent mixed‑methods study design (4). However, we did not find any major discrepancies or contradictions, considering the results were strongly in favour of the virtual simulation, and this somewhat facilitated the integration of the results. |
| 1. Describe any insights gained from mixing or integrating methods. | pp.12-13).  The use of the quantitative method made it possible first to measure a great number of items on the simulation acceptability and then to obtain a descriptive picture of nurses’ perception of the acceptability of this educational intervention. However, the quantitative data did not lead to understanding nurses’ learning experience—a gap that justified the use of a qualitative component. The added value of this latter was to nuance and provide an understanding of the simulation experience, particularly with respect to its usefulness for learning. The insights of combining methods allowed us to connect and provoke “interactions” between the simulation’s features and elements and their contribution to learning progression. By integrating the two components, we highlighted the convergence of the results. They were not surprising, considering the high averages and high level of agreement in favour of the simulation in the quantitative results that were subsequently supported by the transcript/themes. |

**References**

1. O'Cathain A, Murphy E, Nicholl J. The Quality of Mixed Methods Studies in Health Services Research. J Health Serv Res Policy. 2008;13(2):92-8.

2. Sidani S, Braden CJ. Testing the Acceptability and Feasibility of Interventions. In: Sidani S, Braden CJ, editors. Design, Evaluation, and Translation of Nursing Interventions: John Wiley & Sons, Ltd; 2011. p. 163-96.

3. Bryman A. Integrating quantitative and qualitative research: how is it done? Qual Res. 2006;6(1):97-113.

4. Creswell JW, Creswell JD. Research design: Qualitative, quantitative, and mixed methods approaches. 5 ed. Los Angeles, CA: Sage Publications; 2018.

5. Pluye P, Bengoechea EG, Granikov V, Kaur N, Tang DL. A World of Possibilities in Mixed Methods: Review of the Combinations of Strategies Used to Integrate Qualitative and Quantitative Phases, Results and Data. IJMRA. 2018;10(1):41–56.

6. Johnson RE, Grove AL, Clarke A. Pillar Integration Process: A Joint Display Technique to Integrate Data in Mixed Methods Research. J Mix Methods Res. 2019;13(3):301-20.

7. Johnson RB, Onwuegbuzie AJ. Mixed Methods Research: A Research Paradigm Whose Time Has Come. Educ Res. 2004;33(7):14-26.

8. Chicoine G. How are we conducting and reporting mixed methods research in nursing sciences? A commentary on Seah et al. (2018) publication. Arch Psychiatr Nurs. 2018;32(6):784-5.

9. Cheng Y-M. The effects of information systems quality on nurses’ acceptance of the electronic learning system. J Nurs Res. 2012;20(1):19-31.
