# Supplementary material for: Virtual patient simulation to improve nurses’ relational skills in a continuing education context: a convergent mixed methods study
Source: BMC Nurs. 2022 Jan 4;21:1. doi: 10.1186/s12912-021-00740-x (PMC8725454; doi:10.1186/s12912-021-00740-x)
Supplement: Supplementary file 7 — Additional file 7. VP simulation design elements – Quantitative findings. [file 12912_2021_740_MOESM7_ESM.docx]

Additional file 7. Virtual patient simulation design elements

| Items | m (SD)^a^ | Med (IQR)^b^ | 1 - Strongly disagree n (%)^c^ | 2 - Disagree  n (%) | 3 - Agree  n (%) | 4 - Strongly agree  n (%) | N/A^d^ |
| --- | --- | --- | --- | --- | --- | --- | --- |
| CONTEXT OF THE VIRTUAL PATIENT SIMULATION/ PREBRIEFING |  |  |  |  |  |  |  |
| The student-researcher’s animated video introduction was key to fully understanding the context of the virtual simulation. | 3.44 (0.77) | 4 (1) | 1 (4) | 1 (4) | 9 (33) | 14 (52) | 2 (7) |
| The textual introduction (which repeated the same content as the video introduction) was key to fully understanding the context of the virtual simulation. | 3.14 (0.73) | 3 (1) | 0 (0) | 4 (15) | 10 (37) | 7 (26) | 6 (22) |
| I think it is important to have access to both information formats (text and video) to understand the context of the virtual simulation. | 3.15 (0.6) | 3 (0.5) | 0 (0) | 3 (11) | 17 (63) | 7 (26) | 0 (0) |
| GLOSSARY |  |  |  |  |  |  |  |
| The glossary was useful to my learning. | 2.91 (0.79) | 3 (0.5) | 1 (4) | 5 (19) | 12 (44) | 5 (19) | 4 (15) |
| The glossary is a key resource for complementary information about the whole nurse-patient consultation (including quizzes and feedback). | 2.96 (0.88) | 3 (0.5) | 2 (7) | 3 (11) | 12 (44) | 6 (22) | 4 (15) |
| The glossary contained technical terms that were difficult for me to understand. (*negative item*) | 1.74 (0.75) | 2 (1) | 9 (33) | 12 (44) | 1 (4) | 1 (4) | 4 (15) |
| I intend to use the glossary as a reference document in the future. | 2.89 (0.97) | 3 (0.5) | 4 (15) | 2 (7) | 14 (52) | 7 (26) | 0 (0) |
| ELECTRONIC PATIENT RECORD |  |  |  |  |  |  |  |
| I received enough information in all the different sections of the “Patient Record” to fully understand the patient’s situation. | 3.56 (0.58) | 4 (1) | 0 (0) | 1 (4) | 10 (37) | 16 (59) | 0 (0) |
| QUIZZES |  |  |  |  |  |  |  |
| The quizzes required I take time to reflect before choosing my answers. | 2.93 (0.68) | 3 (0) | 2 (7) | 1 (4) | 21 (78) | 3 (11) | 0 (0) |
| I saw myself in some of the quiz answers. | 3.37 (0.49) | 3 (1) | 0 (0) | 0 (0) | 17 (63) | 10 (37) | 0 (0) |
| The quizzes made me reflect on my nursing practice. | 3.48 (0.51) | 3 (1) | 0 (0) | 0 (0) | 14 (52) | 13 (48) | 0 (0) |
| There were a sufficient number of quizzes. | 3.33 (0.62) | 3 (1) | 0 (0) | 2 (7) | 14 (52) | 11 (41) | 0 (0) |
| FEEDBACK |  |  |  |  |  |  |  |
| The feedback allowed me to make the connections between the simulated situation and the theoretical elements of MI. | 3.52 (0.51) | 4 (1) | 0 (0) | 0 (0) | 13 (48) | 14 (52) | 0 (0) |
| Feedback was provided in a timely manner (as the consultation progressed). | 3.59 (0.5) | 4 (1) | 0 (0) | 0 (0) | 11 (41) | 16 (59) | 0 (0) |
| Getting the feedback right after the quizzes was disruptive to my learning. (*negative item*) | 1.41 (0.57) | 1 (1) | 17 (63) | 9 (33) | 1 (4) | 0 (0) | 0 (0) |
| I would have preferred to get the feedback at the end of the interview. | 1.33 (0.55) | 1 (1) | 19 (70) | 7 (26) | 1 (4) | 0 (0) | 0 (0) |
| I would have liked to have been able to select the format of feedback (audio and/or text). | 1.89 (0.75) | 2 (1) | 8 (30) | 15 (56) | 3 (11) | 1 (4) | 0 (0) |
| VISUAL CUES/LABELS |  |  |  |  |  |  |  |
| I found that the green and red labels next to the dialogue constructively supported my learning. | 3.48 (0.51) | 3 (1) | 0 (0) | 0 (0) | 14 (52) | 13 (48) | 0 (0) |
| Red or green labels were key to qualifying the content of the nurse-patient dialogue. | 3.33 (0.55) | 3 (1) | 0 (0) | 1 (4) | 16 (59) | 10 (37) | 0 (0) |
| FIDELITY |  |  |  |  |  |  |  |
| The story of the virtual patient who had difficulty following his treatment was realistic. | 3.56 (0.58) | 4 (1) | 0 (0) | 1 (4) | 10 (37) | 16 (59) | 0 (0) |
| The environment in which the interview took place resembled a nurse’s office. | 3.33 (0.55) | 3 (1) | 0 (0) | 1 (4) | 16 (59) | 10 (37) | 0 (0) |
| The virtual patient’s appearance resembled of a typical man living with HIV. | 3.07 (0.73) | 3 (0.5) | 1 (4) | 3 (11) | 16 (59) | 7 (26) | 0 (0) |
| Virtual simulation realistically reproduced nurse-patient interactions. | 3.26 (0.59) | 3 (1) | 0 (0) | 2 (7) | 16 (59) | 9 (33) | 0 (0) |

^a^ m: mean, SD: standard deviations calculated on 4-point Likert scale (range: 1−4).

^b:^ med: median, IQR: interquartile range.

^c:^  Results presented as categorical variable. n: number of participants and % of participants.

^d^ N/A: not applicable means “I did not watch or consult it.” Some virtual patient simulation design elements, such as the context (prebriefing video and text), the glossary and the patient’s electronic record, were not mandatory. In brackets (% of response)
