# Supplementary material for: Virtual patient simulation to improve nurses’ relational skills in a continuing education context: a convergent mixed methods study
Source: BMC Nurs. 2022 Jan 4;21:1. doi: 10.1186/s12912-021-00740-x (PMC8725454; doi:10.1186/s12912-021-00740-x)
Supplement: Supplementary file 8 — Additional file 8. Techno acceptance – Quantitative findings based on the Technology Acceptance Model. [file 12912_2021_740_MOESM8_ESM.docx]

Additional file 8 Global system quality and technology acceptance

| **dimensions**  **Constructs**  Items | Cron-bach’s  alpha | m (SD) | Med (IQR) | 1 –  Strongly disagree  n (%) | 2-  Disagree  n (%) | 3-  Agree  n (%) | 4 - Strongly  agree  n (%) | N/A^a^  n (%) |
| --- | --- | --- | --- | --- | --- | --- | --- | --- |
| **Global system quality** ^b^ |  | 3.53 (0.53) | 4 (1) |  |  |  |  |  |
| **System quality** | 0.83 | 3.51 (0.54) | 4 (1) |  |  |  |  |  |
| The virtual simulation can gave learners control over their learning activity. |  | 3.41 (0.57) | 3 (1) | 0 (0) | 1 (4) | 14 (52) | 12 (44) | 0 (0) |
| The virtual simulation presented course materials in a multimedia and readable format. |  | 3.44 (0.51) | 3 (1) | 0 (0) | 0 (0) | 15 (56) | 12 (44) | 0 (0) |
| The virtual simulation can offer flexibility in learning as to time. |  | 3.63 (0.49) | 4 (1) | 0 (0) | 0 (0) | 10 (37) | 17 (63) | 0 (0) |
| The virtual simulation can offer flexibility in learning as to place. |  | 3.48 (0.64) | 4 (1) | 0 (0) | 2 (7) | 10 (37) | 15 (56) | 0 (0) |
| The digital simulation was interactive. |  | 3.59 (0.5) | 4 (1) | 0 (0) | 0 (0) | 11 (41) | 16 (59) | 0 (0) |
| **Information quality** | 0.86 | 3.49 (0.50) | 3 (1) |  |  |  |  |  |
| The content of the virtual simulation was innovative. |  | 3.56 (0.51) | 4 (1) | 0 (0) | 0 (0) | 12 (44) | 15 (56) | 0 (0) |
| The virtual simulation met my learning needs. |  | 3.48 (0.51) | 3 (1) | 0 (0) | 0 (0) | 14 (52) | 13 (48) | 0 (0) |
| The level of difficulty of the virtual simulation learning content was appropriate. |  | 3.44 (0.51) | 3 (1) | 0 (0) | 0 (0) | 15 (56) | 12 (44) | 0 (0) |
| **Service quality** | 1.00^c^ | 3.65 ^d^ (0.48) | 4 (1) |  |  |  |  |  |
| I acquired adequate support within the virtual simulation to help my learning (e.g. user guide). |  | 3.67^e^ (0.49) | 4 (1) | 0 (0) | 0 (0) | 5 (19) | 10 (37) | 12 (44) |
| I acquired adequate support from the virtual simulation’s administrators for the app’s technical aspects. |  | 3.73^f^ (0.47) | 4 (0.5) | 0 (0) | 0 (0) | 3 (11) | 8 (30) | 16 (59) |
| Overall, support services of the virtual simulation were satisfactory. |  | 3.57^g^ (0.51) | 4 (1) | 0 (0) | 0 (0) | 6 (22) | 8 (30) | 13 (48) |
| **User-interface design quality** | 0.68 | 3.54 (0.55) | 4 (1) |  |  |  |  |  |
| The layout of the virtual simulation was user friendly. |  | 3.37 (0.56) | 3 (1) | 0 (0) | 1 (4) | 15 (56) | 11 (41) | 0 (0) |
| The layout of the virtual simulation was well structured. |  | 3.89 (0.32) | 4 (0) | 0 (0) | 0 (0) | 3 (11) | 24 (89) | 0 (0) |
| Overall, user-interface design of the virtual simulation was satisfactory (e.g. overall design, 3D images, colours, consultation processes). |  | 3.37 (0.56) | 3 (1) | 0 (0) | 1 (4) | 15 (56) | 11 (41) | 0 (0) |
| **Technology acceptance**^a^ |  | 3.45 (0.64) | 4 (1) |  |  |  |  |  |
| **Perceived usefulness** | 0.92 | 3.35 (0.71) | 3 (1) |  |  |  |  |  |
| Using the virtual simulation seemed to me to be more effective than other types of training I might have received. |  | 3.33 (0.78) | 4 (1) | 0 (0) | 5 (19) | 8 (30) | 14 (52) | 0 (0) |
| Using the virtual simulation enhanced the effectiveness of my learning. |  | 3.26 (0.81) | 3 (1) | 0 (0) | 6 (22) | 8 (30) | 13 (48) | 0 (0) |
| I found the virtual simulation to be useful in my learning. |  | 3.44 (0.51) | 3 (1) | 0 (0) | 0 (0) | 15 (56) | 12 (44) | 0 (0) |
| **Perceived ease of use** | 0.69 | 3.42 (0.67) | 4 (1) |  |  |  |  |  |
| Using virtual simulation did not require a lot of mental effort. |  | 3.22 (0.8) | 3 (1) | 1 (4) | 3 (11) | 12 (44) | 11 (41) | 0 (0) |
| I have found the virtual simulation to be easy to use. |  | 3.48 (0.64) | 4 (1) | 0 (0) | 2 (7) | 10 (37) | 15 (56) | 0 (0) |
| I quickly developed ease in using the virtual simulation. |  | 3.56 (0.51) | 4 (1) | 0 (0) | 0 (0) | 12 (44) | 15 (56) | 0 (0) |
| **Perceived enjoyment** | 0.92 | 3.47 (0.57) | 4 (1) |  |  |  |  |  |
| I have found using the virtual simulation enjoyable. |  | 3.52 (0.58) | 4 (1) | 0 (0) | 1 (4) | 11 (41) | 15 (56) | 0 (0) |
| The navigation within the virtual simulation was pleasant. |  | 3.37 (0.56) | 3 (1) | 0 (0) | 1 (4) | 15 (56) | 11 (41) | 0 (0) |
| I have had fun using the virtual simulation. |  | 3.52 (0.58) | 4 (1) | 0 (0) | 1 (4) | 11 (41) | 15 (56) | 0 (0) |
| **Intention to use** | 0.96 | 3.53 (0.60) | 4 (1) |  |  |  |  |  |
| I would use the virtual simulation on a regular basis in the future, if it were available online. |  | 3.52 (0.64) | 4 (1) | 0 (0) | 2 (7) | 9 (33) | 16 (59) | 0 (0) |
| I would use the virtual simulation frequently in my practice, if it were available online. |  | 3.44 (0.64) | 4 (1) | 0 (0) | 2 (7) | 11 (41) | 14 (52) | 0 (0) |
| I would strongly recommend the virtual simulation be made available to other nurses. |  | 3.56 (0.58) | 4 (1) | 0 (0) | 1 (4) | 10 (37) | 16 (59) | 0 (0) |
| I would strongly recommend the virtual simulation be made available to other healthcare professionals. |  | 3.59 (0.57) | 4 (1) | 0 (0) | 1 (4) | 9 (33) | 17 (63) | 0 (0) |

^a^ N/A: not applicable

^b^ For the two dimensions, global system quality and technology acceptance, and their corresponding constructs, only the means and standard deviation are presented. The sum of categorical variables would be confusing because the n would become higher than the numbers of participants (n=27).

^c^ The Cronbach alpha was calculated for participants (n=9) who answered the three items of the constructs. Otherwise, the descriptive statistics for each item were calculated per response (or entry).

^d^ 41 entries were answered “not applicable,” representing 16 participants; this means that these did not require any service/support during their participation in the VP simulation.

^e^ 12 entries were answered “not applicable” (n=12 participants)

^f^ 16 entries were answered “not applicable”(n=16 participants)

^g^ 13 entries were answered “not applicable” (n=13 participants)
