# Supplementary material for: Virtual patient simulation to improve nurses’ relational skills in a continuing education context: a convergent mixed methods study
Source: BMC Nurs. 2022 Jan 4;21:1. doi: 10.1186/s12912-021-00740-x (PMC8725454; doi:10.1186/s12912-021-00740-x)
Supplement: Supplementary file 9 — Additional file 9. Simulation’ role to support practice – Quantitative findings. [file 12912_2021_740_MOESM9_ESM.docx]

Additional file 9. The role of simulation in supporting nurses’ professional practice

| Items | m (SD) | Med (IQR) | 1 - Strongly disagree  n (%) | 2 –  Disagree  n(%) | 3 –  Agree  n(%) | 4 – Strongly Agree  n(%) | N/A^b^  n (%) |
| --- | --- | --- | --- | --- | --- | --- | --- |
| The virtual simulation led me to reflect on my nursing practice in the overall healthcare context offered to PLHIV.^a^ | 3.45 (0.51) | 3 (1) | 0 (0) | 0 (0) | 11 (41) | 9 (33) | 7 (26) |
| The virtual simulation led me to reflect on my nursing practice as part of antiretroviral treatment support. | 3.55 (0.51) | 4 (1) | 0 (0) | 0 (0) | 9 (33) | 11 (41) | 7 (26) |
| Integration of teaching assisted by the virtual simulation will allow me to improve my communication skills with PLHIV. | 3.45 (0.51) | 3 (1) | 0 (0) | 0 (0) | 11 (41) | 9 (33) | 7 (26) |
| Integration of teaching assisted by virtual simulation will allow me to improve the health of PLHIV. | 3.50 (0.51) | 3.5 (1) | 0 (0) | 0 (0) | 10 (37) | 10 (37) | 7 (26) |
| Integration of teaching assisted by virtual simulation will allow me to improve the quality of therapeutic relationships with PLHIV. | 3.5 (0.51) | 3.5 (1) | 0 (0) | 0 (0) | 10 (37) | 10 (37) | 7 (26) |
| I feel capable of applying the communication skills seen in the virtual simulation to PLHIV. | 3.38 (0.5) | 3 (1) | 0 (0) | 0 (0) | 13 (48) | 8 (30) | 6 (22) |
| My participation in the virtual simulation has made me more confident about facing similar situations with others PLHIV. | 3.29 (0.56) | 3 (1) | 0 (0) | 1 (4) | 13 (48) | 7 (26) | 6 (22) |
| The virtual simulation led me to reflect about my nursing practice in general, not just with PLHIV. | 3.58 (0.58) | 4 (1) | 0 (0) | 1 (4) | 9 (33) | 16 (59) | 1 (4) |
| Integration of teaching assisted by virtual simulation will lead me to improve my communication skills with clientele other than PLHIV. | 3:50 (0.51) | 3.5 (1) | 0 (0) | 0 (0) | 13 (48) | 13 (48) | 1 (4) |
| Integration of teaching assisted by virtual simulation will allow me to improve the health of other clientele than PLHIV. | 3.38 (0.57) | 3 (1) | 0 (0) | 1 (4) | 14 (52) | 11 (41) | 1 (4) |
| Integration of teaching assisted by virtual simulation will lead me to improve the quality of therapeutic relationship with other clientele than PLHIV. | 3.33 (0.62) | 3 (1) | 0 (0) | 2 (7) | 14 (52) | 11 (41) | 0 (0) |
| I feel capable of applying the communication skills seen in virtual simulation to other clientele than PLHIV. | 3:30 (0.54) | 3 (1) | 0 (0) | 1 (4) | 17 (63) | 9 (33) | 0 (0) |
| My participation in the virtual simulation has made me more confident about facing similar situations with clientele other than PLHIV. | 3.26 (0.59) | 3 (1) | 0 (0) | 2 (7) | 16 (59) | 9 (33) | 0 (0) |
| I learned from the mistakes I made in the virtual simulation. | 3.37 (0.49) | 3 (1) | 0 (0) | 0 (0) | 17 (63) | 10 (37) | 0 (0) |
| As a result of my virtual simulation, I have identified certain aspects of my professional practice that I could improve. | 3.22 (0.42) | 3 (0) | 0 (0) | 0 (0) | 21 (78) | 6 (22) | 0 (0) |
| I learned something new by participating in this virtual simulation. | 3.48 (0.51) | 3 (1) | 0 (0) | 0 (0) | 14 (52) | 13 (48) | 0 (0) |
| Integration of teaching assisted by virtual simulation will allow me to increase the use of change talk. | 3.19 (0.4) | 3 (0) | 0 (0) | 0 (0) | 22 (81) | 5 (19) | 0 (0) |
| Integration of teaching assisted by virtual simulation will allow me to decrease the use of sustain talk. | 2.89 (0.64) | 3 (0) | 1 (4) | 4 (15) | 19 (70) | 3 (11) | 0 (0) |
| The virtual simulation raised my awareness of elements that can facilitate therapeutic relationships with patients. | 3.22 (0.42) | 3 (0) | 0 (0) | 0 (0) | 21 (78) | 6 (22) | 0 (0) |
| The virtual simulation has made me aware of the “traps” that can make therapeutic relationships with patients difficult. | 3.30 (0.47) | 3 (1) | 0 (0) | 0 (0) | 19 (70) | 8 (30) | 0 (0) |
| My participation in teaching assisted by the virtual simulation has helped me understand how the theoretical notions (from MI^c^) could be applied in my practice. | 3.22 (0.51) | 3 (0.5) | 0 (0) | 1 (4) | 19 (70) | 7 (26) | 0 (0) |
| My participation in teaching assisted by the virtual simulation has been a useful learning experience for my continuing professional development. | 3.33 (0.48) | 3 (1) | 0 (0) | 0 (0) | 18 (67) | 9 (33) | 0 (0) |

^a^ PLHIV: people living with HIV

^b^ N/A: not applicable

^c^ MI: motivational interviewing
