# Supplementary material for: Virtual patient simulation to improve nurses’ relational skills in a continuing education context: a convergent mixed methods study
Source: BMC Nurs. 2022 Jan 4;21:1. doi: 10.1186/s12912-021-00740-x (PMC8725454; doi:10.1186/s12912-021-00740-x)
Supplement: Supplementary file 10 — Additional file 10. Achievement learning objectives – Quantitative findings. [file 12912_2021_740_MOESM10_ESM.docx]

Additional file 10. Achievement of learning objectives

| Items  (The virtual simulation make it possible to…) | m (SD) | Med (IQR) | 1 –  Strongly disagree  n (%) | 2 –  Disagree  n(%) | 3 –  Agree  n(%) | 4 –  Strongly agree  n(%) | N/A^a^  n(%) |
| --- | --- | --- | --- | --- | --- | --- | --- |
| Spot traps in nursing interventions that can shut down communication with the patient. | 3.58 (0.5) | 4 (1) | 0 (0) | 0 (0) | 11 (41) | 15 (56) | 1 (4) |
| Identify nursing interventions that optimize openness to the patient’s experience. | 3.54 (0.51) | 4 (1) | 0 (0) | 0 (0) | 12 (44) | 14 (52) | 1 (4) |
| Apply nursing interventions that elicit change talk. | 3.48 (0.51) | 3 (1) | 0 (0) | 0 (0) | 14 (52) | 13 (48) | 0 (0) |
| Spot traps in nursing interventions that enable the statu quo (sustain talk). | 3.41 (0.5) | 3 (1) | 0 (0) | 0 (0) | 16 (59) | 11 (41) | 0 (0) |
| Identify cues in the patient’s speech that reflect change talk. | 3.42 (0.5) | 3 (1) | 0 (0) | 0 (0) | 15 (56) | 11 (41) | 1 (4) |
| Target the key elements that are important to include in providing information to the patient. | 3.37 (0.56) | 3 (1) | 0 (0) | 1 (4) | 15 (56) | 11 (41) | 0 (0) |
| Identify principles to build an action plan with the patient. | 3.38 (0.5) | 3 (1) | 0 (0) | 0 (0) | 16 (59) | 10 (37) | 1 (4) |
| Describe the principles consistent with MI that structure information sharing with the patient. | 3.35 (0.49) | 3 (1) | 0 (0) | 0 (0) | 17 (63) | 9 (33) | 1 (4) |

^a^ N/A: Not applicable means, “I already mastered the topic.”.
