# Supplementary material for: Virtual patient simulation to improve nurses’ relational skills in a continuing education context: a convergent mixed methods study
Source: BMC Nurs. 2022 Jan 4;21:1. doi: 10.1186/s12912-021-00740-x (PMC8725454; doi:10.1186/s12912-021-00740-x)
Supplement: Supplementary file 11 — Additional file 11. Operationalization integration – Application of the pillar integration process and an excerpt of the joint display. [file 12912_2021_740_MOESM11_ESM.docx]

Additional file 11. Operationalization of the pillar integration process and excerpt of the joint display

*Stage 1 – Listing:* Selecting the relevant raw quantitative data to be listed in the column (QUANT data). One example of a QUANT data was: “*The story of the virtual patient who had difficulty following his treatment was realistic* (3.56±0.58)”. This data belongs to the “fidelity” QUANT category.

*Stage 2 – Matching:* Listing QUAL data (verbatim) and categories (themes) in their respective columns to match the QUANT data and categories columns. One QUAL data gained from the focus group that matched the example provided in *Stage 1* was: “*The patient’s story was really, um, it’s real life, it’s really believable; it’s not something just pulled out of thin air*.” This QUAL data supported the QUAL category (theme) as follows: Enjoying the practice in a realistic, immersive and non-judgmental environment.

*Stage 3 – Checking:* Cross-checking the QUANT and QUAL data/categories and their emerging patterns (or lack thereof), for quality-integration purposes.

*Stage 4 – Pillar building:* Comparing, contrasting, integrating and connecting both types of data and categories from the listing, matching, and checking phases to build inferences. Writing a meaningful narrative to relate mixed evidence with a weaving approach and grouping quantitative and qualitative findings under mixed method interpretations. Based on the examples provided in Stage 1 and Stage 2, we ended up with this mixed method interpretation finding (see “Pillar building with mixed method interpretations” column): “*Influence of the simulation’s fidelity on nurses’ impression of getting real practice and of having an immersive learning experience*.”

Table 1. Excerpt of the joint display used as part of the pillar integration process

| **QUANT** ^a^ **data** | **QUANT categories** | **Pillar building with mixed method interpretations** | **QUAL categories (themes)** | **QUAL** ^b^ **data – Verbatim** |
| --- | --- | --- | --- | --- |
|  | |  |  |  |
| Not identified | Not  identified | Not identified because this is a new theme that came from the qualitative component | Theme: Motivations to engage in the simulation-based research | There was nevertheless a link between the patients (with hepatitis C) adhering to the treatment I’m following here and the motivational talk with patients with drug addiction. I could see the sense in it. (Male assistant head nurse, FG^c^)  I don’t really see any (patients with) HIV, but we do youth clinics, so we have screening. I found it important to do this training to learn things about HIV but also about motivational interviewing, which we do daily, enormously, at our office. (Female school nurse, FG) |
| The story of the virtual patient who had difficulty following his treatment was realistic. (3.56±0.58) | Fidelity | Influence of the simulation’s fidelity on nurses’ impression of getting real practice and of having an immersive learning experience | Theme: Learning in a realistic, immersive, and non-judgmental environment | The patient’s story was really, um, it’s real life, it’s really believable; it’s not something just pulled out of thin air. (Male head assistant nurse, FG)  Maybe this is because I’ve done a lot of work around the issue of taking antiretroviral treatment, so I found the [VP’s ^d^] situation ... maybe less typical. (Female nurse-researcher, FG). |
| Virtual simulation realistically reproduced nurse-patient interactions (3.26±0.59) | Fidelity | Influence of the simulation’s fidelity on nurses’ impression of getting real practice and of having an immersive learning experience | Theme: Learning in a realistic, immersive, and non-judgmental environment | It’s virtual but at the same time there was an interaction, which was fairly realistic. I wonder if, even if there had been no character but only the audio—honestly, I was really “in” the situation; I was in all of it. It was very well performed—the voices and all. I found it super well done. (Male assistant head nurse, FG) |
| The feedback allowed me to make the connections between the simulated situation and the theoretical elements of MI^e^. (3.52 ±0.51) | Feedback | Acquiring new knowledge and building self-confidence | Theme: Perceived utility of the virtual patient simulation  Sub-theme: Acquiring and consolidating motivational interviewing knowledge and skills | I found that the platform allowed me to consolidate my nursing practice and my past theoretical learning, since I don’t see patients every day. (Female nurse-manager, FG)  I felt that, toward the end (of the simulation), I had gotten better. I had probably internalized the theory. (Male nurse case manager, FG)  I felt it allowed me to better understand (MI); it’s kind of the practical aspect of the real-life approach. (Female nurse-researcher, FG) |
| My participation in teaching assisted by the virtual simulation has helped me understand how the theoretical notions (from MI) could be applied in my practice. (3.22±0.51) | Simulation’s role in supporting nurses’ professional practice |  |  |  |
| The quizzes made me reflect on my nursing practice. (3.48±0.51) | Quizzes | Taping self-awareness and reflection in relational practice | Theme: Perceived utility of the virtual patient simulation  Sub-theme: Developing reflective learning and transferring it to practice | The first time, I made a lot of mistakes because I told myself that I was going to go with my knowledge and experience. The second time, I did it with my new knowledge. It gives you parallel vantage point onto yourself, onto your own beliefs. (Male nurse case manager, FG) |
| I learned from the mistakes I made in the virtual simulation. (3.37±0.49). | Simulation’s role in supporting nurses’ professional practice | Taping self-awareness and reflection in relational practice |  | It was fun because it’s like action/reaction. It was immediately obvious if you asked the question wrong; you could see the effect. I found it interesting because if you took a wrong action, you could get back on track. That way, we could understand why it was a mistake. (Female nurse-manager, FG) |
| The virtual simulation has made me aware of the “traps” that can make therapeutic relationships with patients difficult. (3.30 ±0.47) | Simulation’s role in supporting nurses’ professional practice | Taping self-awareness and reflection in relational practice |  | Do I go too fast sometimes? Telling myself that, well, he didn’t take it [his treatment], that he must have relapsed, always jumping to my conclusions first. Don’t I miss things sometimes, too? I was thinking that maybe now I will be more careful and try to understand the patient’s reasons and stop just saying “Ah, well, he didn’t take it.” (Male assistant head nurse, FG) |
| The virtual simulation raised my awareness of elements that can facilitate therapeutic relationships with patients. (3.22±0.42). | Simulation’s role in supporting nurses’ professional practice | Taping self-awareness and reflection in relational practice | Theme: Perceived utility of the virtual patient simulation  Sub-theme: Being present and revisiting relational skills | I’d say it’s more in the way the questions are asked. It’s really focused on open-ended questions and on solutions that come from the patient. We [nurses] may have solutions, but they have to come from them [the patients]: that’s when they are most effective [...] How can we ask questions that bring out the best in the patient? (Male nurse case manager, FG) |
| The virtual simulation led me to reflect about my nursing practice in general, not just with PLHIV^f^. (3.58±0.58). | Simulation’s role in supporting nurses’ professional practice | Taping self-awareness and reflection in relational practice | Theme: Perceived utility of the virtual patient simulation  Sub-theme: Developing reflective learning and transferring it to practice | Look, if patients don’t react or aren’t motivated, well, maybe it’s because I too am playing a part as the care provider: maybe I am not addressing them in the right way; maybe I am not considering them in their entirety, according to their beliefs and values. (Female nurse-manager, FG)  Doing it interactively leads you to self-reflection: how would you have reacted? You said something spontaneously, and then it made you reflect on what you answered. (Female school nurse, FG) |
| The virtual simulation raised my awareness of elements that can facilitate therapeutic relationships with patients. 3.22 (±0.42) | Simulation’s role in supporting nurses’ professional practice | Taping self-awareness and reflection in relational practice | Theme: Perceived utility of the virtual patient simulation  Sub-theme: Being present and revisiting relational skills | (VP simulation) helps nurses understand or realize that it’s important to listen, to be there in the here and now. (Female nurse-manager, FG) |
| The virtual simulation can offer flexibility in learning as to time.  (3.63±0.49)  The virtual simulation can offer flexibility in learning as to place. (3.48± 0.64)  The virtual simulation can gave learners control over their learning activity. (3.41 ±0.57) | System quality | Simulation’s perceived flexibility, efficacy, and control over one’s learning led to a positive learning experience. | Theme: Learning in a realistic, immersive, and non-judgmental environment | What I find interesting in fact is that you can do it in your living room or at home or at the office, and you can test your wrong answers and see what you get. (Female nurse manager, FG)  I did the whole thing in one go, but I came back later for some aspects that I had understood less clearly or that I had later asked myself questions about. (Female nurse-researcher, FG) |
| Using the virtual simulation seemed to me to be more effective than other types of training I might have received. (3.33±0.78) | Perceived usefulness (technology acceptance) | Simulation’s perceived flexibility, efficacy, and control over one’s learning led to a positive learning experience. | Theme: Learning in a realistic, immersive, and non-judgmental environment | In classic training activities, we practice with a co-worker. I find that quite biased, because we’ve both just learned the theory and we’re trying to apply it. The other person has just learned the same thing, so, in the end, well, we help each other only a little bit. But here, we were faced with a virtual character who is very realistic. I find it even more real than with, say, another trainee. But for people who are shy in groups, [the simulation] is really very accessible and allows them to progress. (Male assistant head nurse, FG) |
| Using the virtual simulation enhanced the effectiveness of my learning. (3.26 ±0.81)  I learned something new by participating in this virtual simulation. (3.48±0.51). | Simulation’s role in supporting nurses’ professional practice | Simulation’s perceived flexibility and efficacy, and control over one’s learning led to a positive learning experience. | Theme: Perceived utility of the virtual patient simulation  Sub-theme: Acquiring and consolidating motivational interviewing knowledge and skills | I’d read a little about MI, but I’d never done any training. I didn’t expect to learn so much in such a short time. (Female nurse-researcher, FG) |
| Not identified | Not identified | Not identified because this is a new theme that emerged in the qualitative component | Theme: Perceived difficulty in engaging in the simulation-based research | I’m not saying the workflow was slow... but maybe that’s why some people didn’t finish the training activity. I’m not saying it was repetitive, but maybe if they felt it was too slow... When the patient talks, he moves his arms around, and sometimes there was a little delay. This was maybe a feeling I had, since I was persistent at first. (Female nurse-manager, FG) |

^a^ QUANT: quantitative

^b^ QUAL: qualitative

^c^ FG: focus group

^d^ VP: virtual patient

^e^ MI: motivational interviewing

^f^ PLHIV: people living with HIV
